# Analysis of genetic diversity and genome-wide association study for drought tolerance related traits in Iranian bread wheat

**DOI:** 10.1186/s12870-023-04416-3

**Published:** 2023-09-15

**Authors:** Ehsan Rabieyan, Mohammad Reza Bihamta, Mohsen Esmaeilzadeh Moghaddam, Hadi Alipour, Valiollah Mohammadi, Kobra Azizyan, Saeideh Javid

**Affiliations:** 1 Department of Agronomy and Plant Breeding, Faculty of Agricultural Sciences and Engineering, University of Tehran, Karaj, Iran; 2 Cereal Department, Seed and Plant Improvement Institute, Agricultural Research, Education and Extension Organization (AREEO), Karaj, Iran; 3 Department of Plant Production and Genetics, Faculty of Agriculture, Urmia University, Urmia, Iran

**Keywords:** Drought tolerance indices, Genome-wide association study, MTAs, SNP markers, Wheat accessions

## Abstract

**Background:**

Drought is most likely the most significant abiotic stress affecting wheat yield. The discovery of drought-tolerant genotypes is a promising strategy for dealing with the world’s rapidly diminishing water resources and growing population. A genome-wide association study (GWAS) was conducted on 298 Iranian bread wheat landraces and cultivars to investigate the genetic basis of yield, yield components, and drought tolerance indices in two cropping seasons (2018–2019 and 2019–2020) under rainfed and well-watered environments.

**Results:**

A heatmap display of hierarchical clustering divided cultivars and landraces into four categories, with high-yielding and drought-tolerant genotypes clustering in the same group. The results of the principal component analysis (PCA) demonstrated that selecting genotypes based on the mean productivity (MP), geometric mean productivity (GMP), harmonic mean (HM), and stress tolerance index (STI) can help achieve high-yield genotypes in the environment. Genome B had the highest number of significant marker pairs in linkage disequilibrium (LD) for both landraces (427,017) and cultivars (370,359). Similar to cultivars, marker pairs on chromosome 4A represented the strongest LD (*r*^2^ = 0.32). However, the genomes D, A, and B have the highest LD, respectively. The single-locus mixed linear model (MLM) and multi-locus random-SNP-effect mixed linear model (mrMLM) identified 1711 and 1254 significant marker-trait association (MTAs) (-log10 *P* > 3) for all traits, respectively. A total of 874 common quantitative trait nucleotides (QTNs) were simultaneously discovered by both MLM and mrMLM methods. Gene ontology revealed that 11, 18, 6, and 11 MTAs were found in protein-coding regions (PCRs) for spike weight (SW), thousand kernel weight (TKW), grain number per spike (GN), and grain yield (GY), respectively.

**Conclusion:**

The results identified rich regions of quantitative trait loci (QTL) on Ch. 4A and 5A suggest that these chromosomes are important for drought tolerance and could be used in wheat breeding programs. Furthermore, the findings indicated that landraces studied in Iranian bread wheat germplasm possess valuable alleles, that are responsive to water-limited conditions. This GWAS experiment is one of the few types of research conducted on drought tolerance that can be exploited in the genome-mediated development of novel varieties of wheat.

**Supplementary Information:**

The online version contains supplementary material available at 10.1186/s12870-023-04416-3.

## Background

Providing 25% of the total proteins and calories in the human diet and food supplies worldwide relies on wheat (*Triticum aestivum* L.) [1–3]. Wheat grain annual production and consumption reach 750 and 735 million tons, respectively [4]. Global climate change adversely affects wheat yield, raising concerns regarding food security in the future. The genetic dissection of agronomic traits that affects yield and stress tolerance is essential to improve wheat yield [5–7]. The Food and Agriculture Organization estimated that of the United Nations 13 million tons of wheat were produced in Iran in 2022, which is over 28% more compared to 10.1 million tons in 2021. The quarterly global Crop Prospects and Food Situation report’s forecast for 2023 production remains the same at 13 million tons. Drought stresses result in approximately over 50% loss in agricultural productivity [8]. Drought is the main concern for crop productivity in most wheat cultivation areas in Iran. In Iran, approximately 6 million hectares of arable lands have been assigned to wheat cultivation in 2020 [8]. Iran is located in a semi-dry region where end-season drought stress severely affects wheat growth [8].

Drought events adversely affect crop productivity by disrupting a wide range of biochemical/physiological functions on a global scale. Water deficit stress is the most severe environmental stress, limiting plant growth and development worldwide [2, 9]. Drought tolerance refers to the capability of plants to grow, develop, and produce a harvestable yield in the absence of water. [10, 11]. Screen and use of the available genetic resources of crop plants can help to alleviate the impact of climate change on agricultural productivity in arid and semi-arid regions [2, 12, 13]. Understanding how plants adapt to drought stress is crucial for developing new and improved methods to increase drought-tolerant plants [14].

Drought tolerance indices (DTIs) have been widely used for identifying compatible genotypes, by evaluating their performance under non-stressed and stressed environments [15, 16]. In the past, the tolerance index (TOL), geometric mean productivity index (GMP), stress tolerance index (STI), and stress susceptibility index (SSI) have all been employed for genotype selection [15]. The advancement of genetics and genomics has made it possible to decipher the genetic elements that control agronomic traits, as well as to determine their chromosomal locations and thus to identify QTLs in crops, including wheat [13, 17].

Wheat breeding programs need to use innovative technologies to explore the genetic basis of complicated quantitative traits [18]. The genome-wide association mapping (GWAS) technique is an efficient approach to dissect the genetic basis of complex traits by genotyping a large number of accessions with multiple single nucleotide polymorphisms (SNPs) and testing the association between SNPs and agronomic traits [19]. GWAS establishes a relationship between genotype and phenotype based on an assumption that linkage disequilibrium (LD) has developed within a population over several generations [20, 21]. The use of association mapping has been successful in evaluating several agronomic characteristics in plants/crops, including alfalfa [22], sorghum [23], maize [24], soybean [25], wheat [8], and rice [26]. A meta-analysis was conducted to identify the most stable QTLs for grain yield (GY) and grain quality traits in wheat [27]. The results revealed that 449 QTLs were successfully projected onto the genetic consensus map which condensed to 100 Meta-QTL (MQTLs) distributed on wheat chromosomes. The QTLs of thousand kernel weight (TKW) were frequently associated with QTLs for GY and grain protein content with co-localization occurring at 55% and 63%, respectively [27]. Meta-QTL analysis for drought tolerance was undertaken in bread wheat to identify consensus and robust MQTLs using 340 known QTLs from 11 earlier studies; accordingly, 13 MQTLs located on 6 chromosomes (1D, 3B, 5A, 6D, 7A, and 7D) were identified, with a maximum of 4 MQTLs on chromosome 5A. The in-silico expression analysis of these 228 cyclic glucans (CGs) allowed the identification of 14 important CGs, with +3 to −8 fold change in expression under drought (relative to normal conditions) in a tolerant cv. named TAM107 [28].

Our study used a collection of agronomic traits and drought tolerance indices between Iranian wheat varieties and landraces to identify significant SNP loci associated with drought-tolerance traits through a GWAS, using the MLM and mrMLM. The study further aimed to explore candidate genes for drought-resistance traits in bread wheat and to better understand the molecular mechanisms of the drought adaptation of bread wheat in order to facilitate the cultivation of drought-tolerant varieties.

## Results

### Phenotypic data summary

**Fig. 1:**
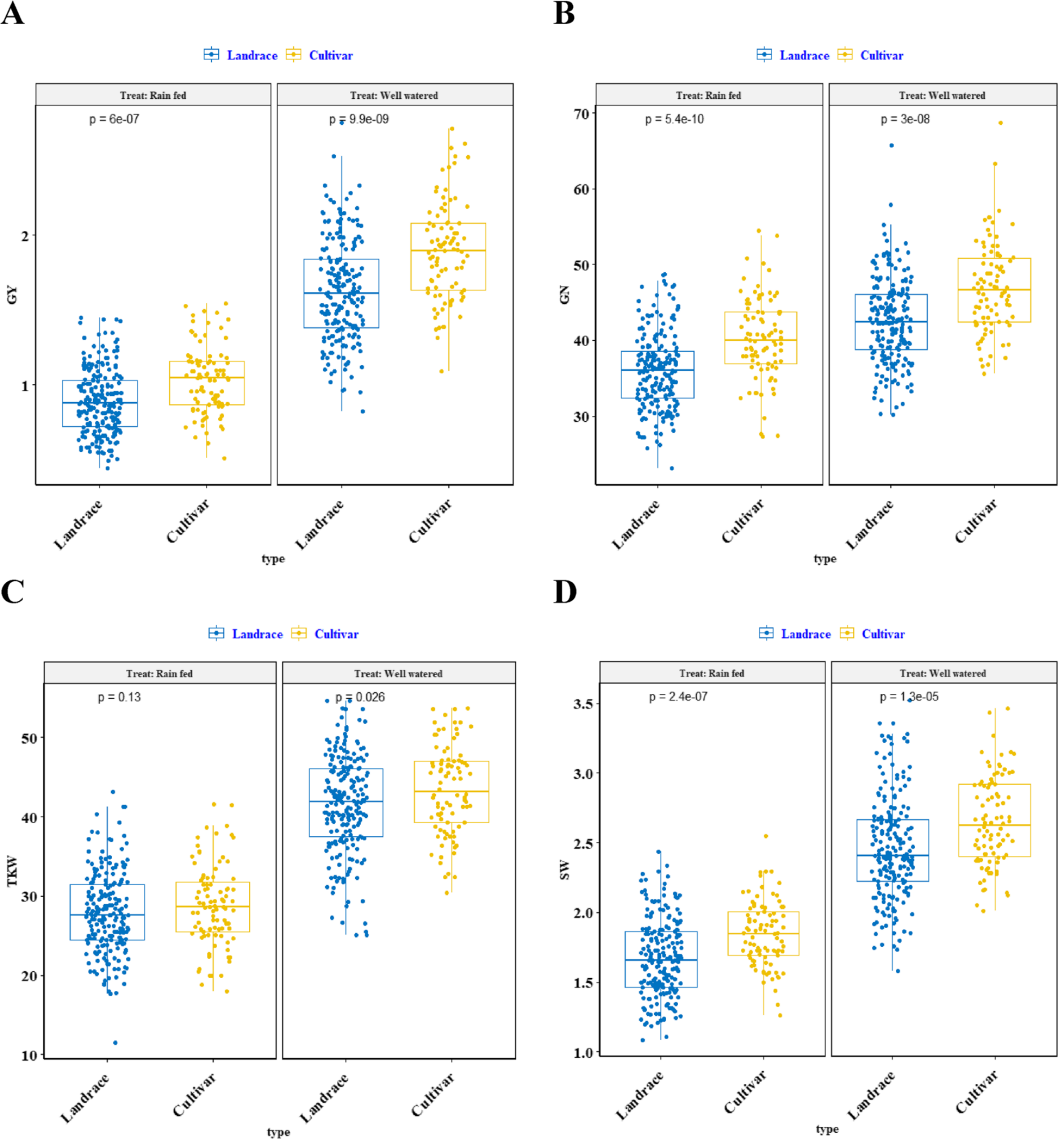
Box-plot representation of the distribution for grain yield (**A**), grains number per spike (**B**), thousand kernel weight (**C**) and spike weight (**D**) for Iranian landraces and cultivars in the well-watered and rain-fed environments

The box plots of wheat landraces and cultivars are displayed in Fig. 1. Compared to a normal situation, the mean of all traits decreased under stress in both cultivars and landraces. Based on the data distribution, there was considerable diversity in wheat accessions regarding agronomical properties. The variance was stronger in native populations. In both moisture conditions, the mean of all traits was lower in landraces than in cultivars.

**Fig. 2:**
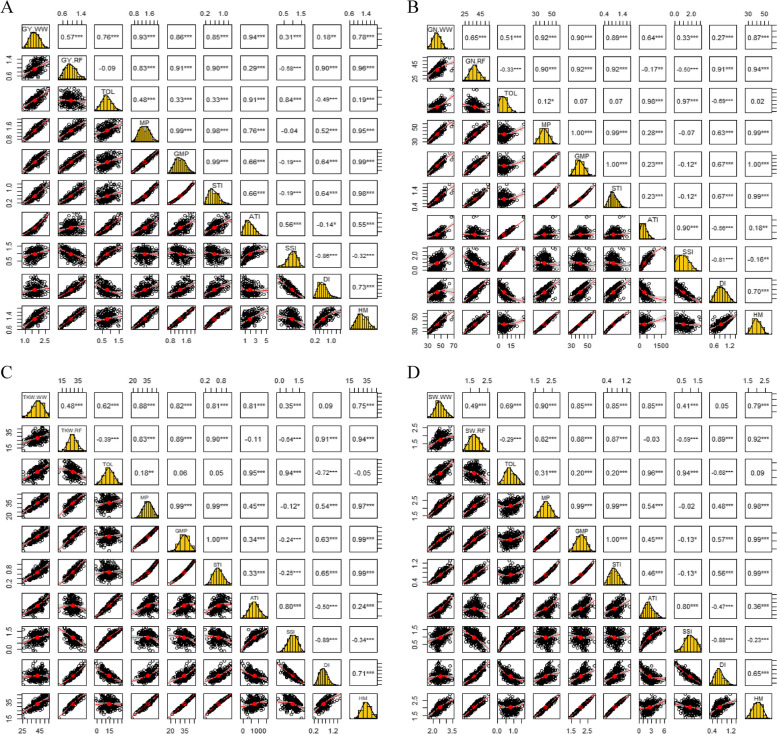
Correlation coefficients between GY_WW_, GY_RF_ and various drought tolerance indices (**A**), GN_WW_, GN_RF_ and various drought tolerance indices (**B**), TKW_WW_, TKW_RF_ and various drought tolerance indices (**C**) and SW_WW_, SW_RF_ and various drought tolerance indices (**D**) for Iranian landraces and cultivars wheat in the well-watered and rain-fed environments. Abbreviations: GY, grain yield; GN, grains number per spike; TKW, thousand kernel weight; SW, spike weight; WW, well-watered; RF, rain-fed; TOL. tolerance index; MP, mean product; GMP, geometric mean product; STI, stress tolerance index; ATI, abiotic stress tolerance index; SSI, stress susceptibility index; DI, new drought resistance index; HM, harmonic mean

The traits GY, GN, TKW, and SW in the normal environment exhibited the highest significant, positive association with the drought tolerance index MP. However, in the rain-fed environment, the mentioned traits had the strongest significant, positive connection with the drought tolerance index HM (Fig. 2).

### Clustering analysis

**Fig. 3:**
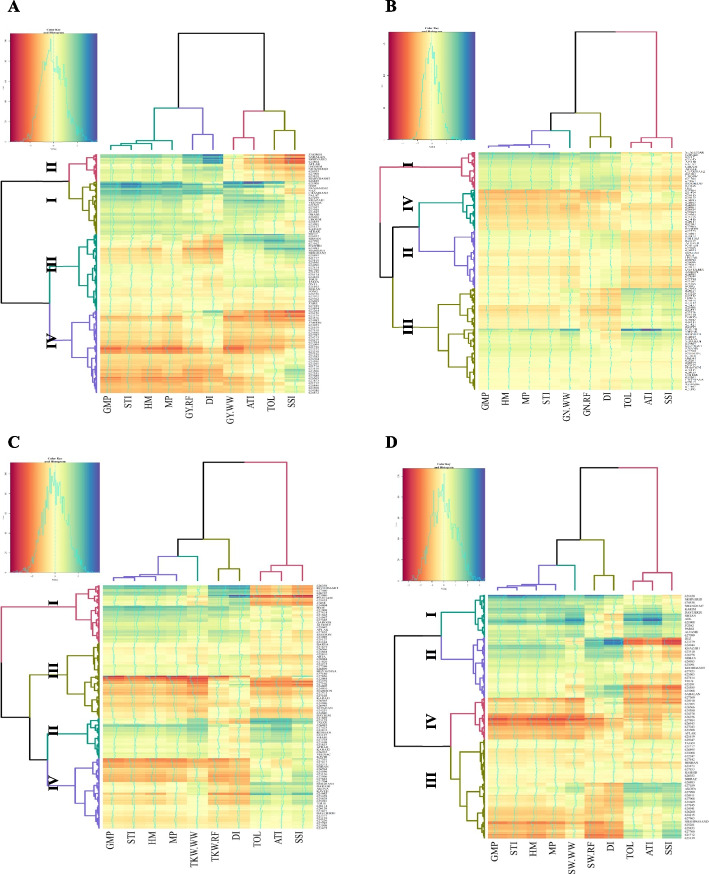
Hierarchical clustering and heatmap based on GY_WW_, GY_RF_ and various drought tolerance indices (**A**), GN_WW_, GN_RF_ and various drought tolerance indices (**B**), TKW_WW_, TKW_RF_ and various drought tolerance indices (**C**) and SW_WW_, SW_RF_ and various drought tolerance indices (**D**) for Iranian landraces and cultivars wheat in well-watered and rain-fed environments. Abbreviations: GY, grain yield; GN, grains number per spike; TKW, thousand kernel weight; SW, spike weight; WW, well-watered; RF, rain-fed; TOL. tolerance index; MP, mean product; GMP, geometric mean product; STI, stress tolerance index; ATI, abiotic stress tolerance index; SSI, stress susceptibility index; DI, new drought resistance index; HM, harmonic mean

The heatmap was created using the average of agronomic features as well as a variety of drought tolerance indices (DTIs). Our purpose in grouping genotypes was to identify genotypes with common characteristics in terms of thousand kernel weight, grains number per spike, spike weight, and grain yield. Heatmap and clustering wheat accessions classified genotypes into four groups based on GY_WW_, GY_RF_, and DTIs (Fig. 3a). Group I contains 66 high-yielding genotypes (32 landraces and 34 cultivars). Further, Group II contains 34 genotypes with average-to-high yields (22 landraces and 12 cultivars), and Group III contains 179 genotypes with average-to-low yields (136 landraces and 43 cultivars). Finally, Group IV contains 19 low-yielding (18 landraces and 1 cultivar). Wheat genotypes were split into four categories using the GN_WW_, GN_RF_, and DTIs. Based on the results, 48 (27 cultivars and 21 landraces), 73 (20 cultivars and 53 landraces), 127 (38 cultivars and 89 landraces), and 50 (5 cultivars and 45 landraces), genotypes were found in the first, second, third, and fourth groups, respectively (Fig. 3b). Clustering and heatmap based on TKW_WW_, TKW_RF_, and DTIs were used to separate wheat accessions into four groups. The first and second groups consisted of 70 genotypes with a high TKW (22 cultivars and 48 landraces), and 49 genotypes with a medium-to-TKW (21 cultivars and 28 landraces), respectively. Moreover, the third and fourth groups contained 93 genotypes with a medium-to-low TKW (68 landraces and 25 cultivars), and 86 genotypes (22 cultivars and 64 landraces) with a low TKW (Fig. 3c), respectively. Wheat genotypes based on the SW_WW_, SW_RF,_ and DTIs were classified into four groups; the I, II, III, and IV groups consisted of 53 (28 landraces and 25 cultivars), 74 (43 landraces and 31 cultivars), 121 (92 landraces and 29 cultivars), and 50 genotypes (45 landraces and 5 cultivars), respectively (Fig. 3d).

### Principal component analysis (PCA) for drought tolerance indices

**Fig. 4:**
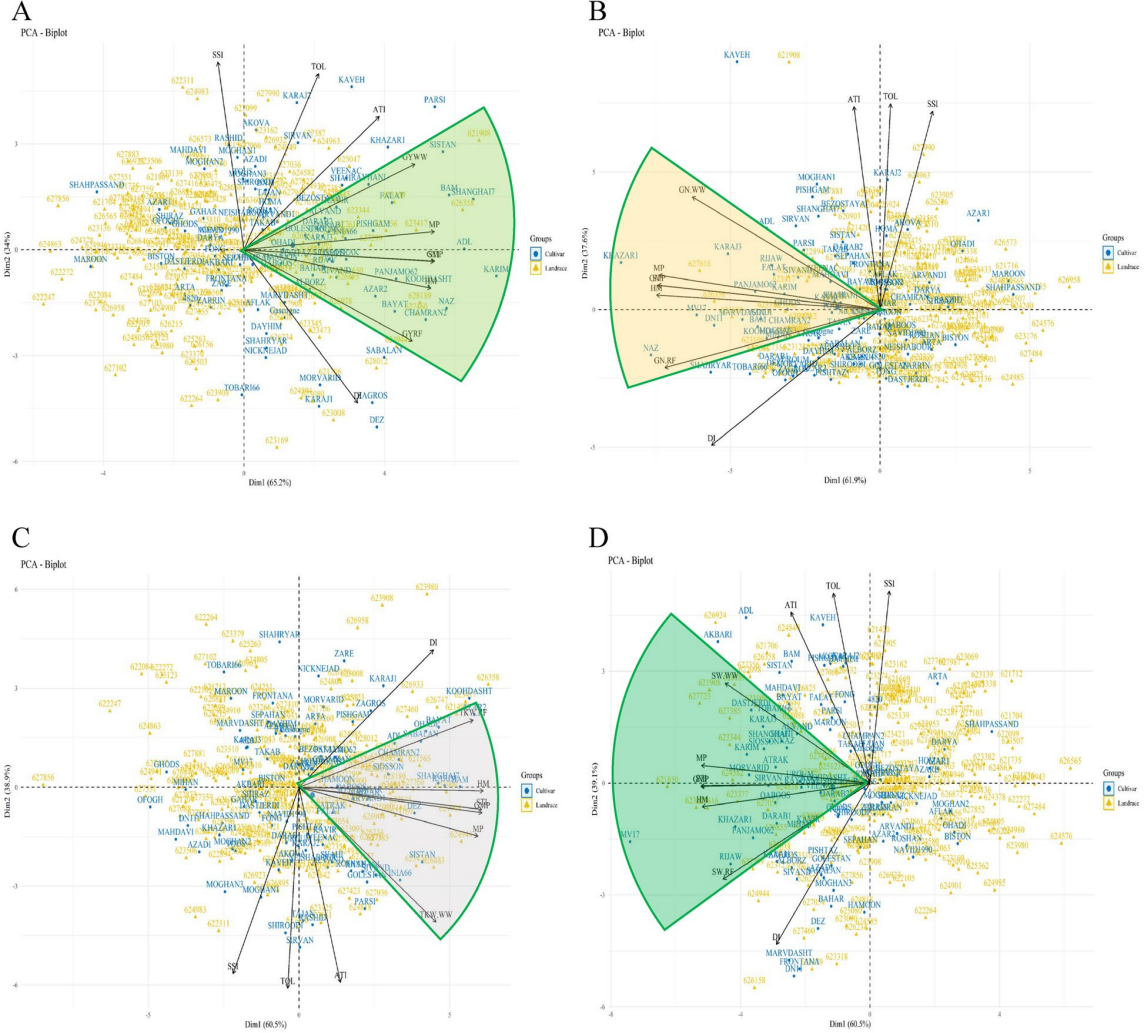
Principal component analysis of Iranian wheat germplasm exposed to well-watered irrigation and rain-fed environments using PC1 and 2. Biplot for GY_WW_, GY_RF_ and various drought tolerance indices (**A**), Biplot for GN_WW_, GN_RF,_ and various drought tolerance indices (**B**), Biplot for TKW_WW_, TKW_RF,_ and various drought tolerance indices (**C**), and Biplot for SW_WW_, SW_RF_ and various drought tolerance indices (**D**). Abbreviations: GY, grain yield; GN, grains number per spike; TKW, thousand kernel weight; SW, spike weight; WW, well-watered; RF, rain-fed; TOL. tolerance index; MP, mean product; GMP, geometric mean product; STI, stress tolerance index; ATI, abiotic stress tolerance index; SSI, stress susceptibility index; DI, new drought resistance index; HM, harmonic mean

For the selection of superior genotypes based on the environment, the responses of all accessions were evaluated with a PCA. The results of PCA for DTIs (based on GY) showed that 65.2% and 34% of the variance were explained by the first and second components, respectively. The attributes GY_WW_, GY_RF_, MP, GMP, STI, and HM had direct associations with PC1. The PC2 had a positive correlation with the SSI, TOL, and ATI indices, whereas the DI index had a negative correlation with the PC2. Genotypes-based PCA revealed that several genotypes (623417, 627299, 628189, 627460, 626855, 622356, 623344, 623109, and 624944, BAM, ADL KARIM, AZARE2, CHAMRAN2, PISHGAM, and FALAT cultivars) had the highest GY in both rain-fed and well-watered environments (Fig. 4a). Nonetheless, selecting genotypes based on the MP, GMP, HM, and STI indices can help achieve high yield genotypes in the environment. The above explanations are also applicable to the other attributes of GN, TKW, and SW (Fig. 4b, c, d).

### Analysis of linked single-nucleotide polymorphisms (SNPs)

**Fig. 5:**
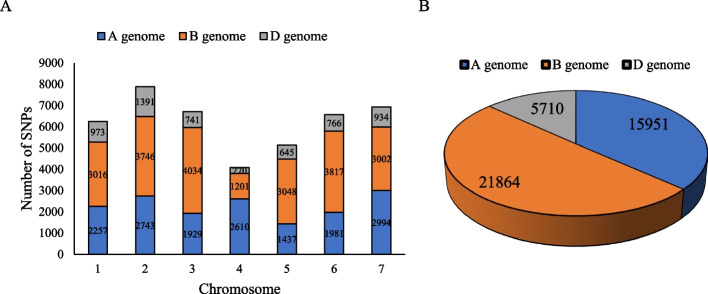
Number of imputed SNPs used in different chromosomes of the wheat genomes (**A**), number of imputed SNPs used in wheat genomes (**B**)

Among the 566,439,207 reads identified in eight Ion Proton runs, 458,363,607 (about 81%) were high-quality barcoded ones. Eventually, 133,039 unique SNPs were identified after removing duplicate reads. A total of 43,525 SNPs were detected across all 21 wheat chromosomes after imputation and discarding those with > 20% missing values, > 10% heterozygosity, and < 5% minor allele frequency. In addition, a set of 43,525 imputed SNPs was obtained using the W7984 reference genome, and these SNPs were used to estimate genetic diversity. Overall 15,951, 21,864, and 5,710 SNPs were mapped to the A, B, and D genomes, respectively, accounting 36.7%, 50.2%, and 13.1% of the total SNPs, respectively (Fig. 5). The highest and lowest numbers of SNPs were located on 3A (4034 SNPs) and 4D (270 SNPs), respectively.

### Population structure and kinship matrix

**Fig. 6:**
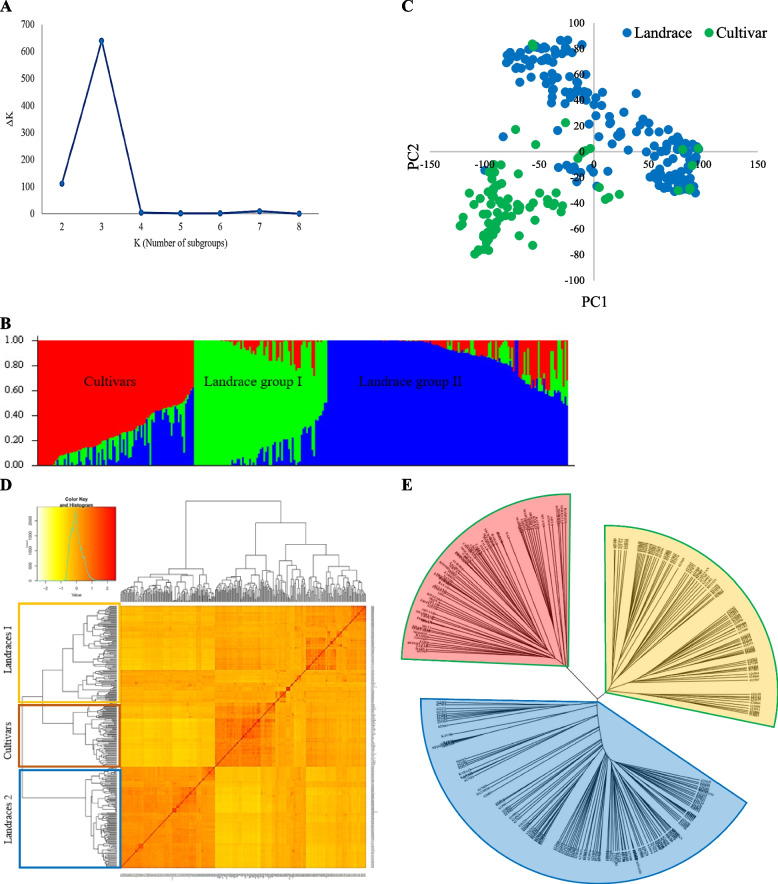
Determination of subpopulations number in wheat genotypes based on ΔK values (**A**), A structure plot of the 298 wheat genotypes and landraces determined by K = 3 (**B**). Principle component analysis (PCA) for a total of 298 Iranian bread wheat accessions (**C**). Cluster analysis using Kinship matrix of imputed data for Iranian wheat accessions (**D**). The dendrogram of Neighbor-Joining clustering constructed using 43,525 SNPs and 298 Iranian wheat accessions (**E**)

The number of clusters (K) and subpopulations (ΔK) was plotted against each other to determine the appropriate number of subpopulations. Three subpopulations were observed as the strongest ΔK value at K = 3. Three subpopulations of 298 accessions were created using the structure software, S-I, S-II, and S-III (Fig. 6a). S_I contained 113 genotypes (107 landraces and 6 cultivars). Furthermore, S_II encompasses 111 genotypes (97 landraces and 14 cultivars), and S_III consists of 74 genotypes (4 landraces and 70 cultivars) (Fig. 6b). According to a PCA based on molecular markers, the first and second components explain 16.9% and 6.3% of the total genetic variance between wheat accessions. This study could distinguish landraces from cultivars favorably. In the Iranian wheat landraces, a population structure was found with 30.5% genetic diversity, accounting for the first five eigenvalues. The selection effects in breeding programs are considered the reasons for such a genetic separation (Fig. 6c). Heatmap analysis using the kinship matrix for Iranian genotypes is illustrated in Fig. 6d. Clustering results identified two subgroups of native populations. Using imputed markers, we could also separate cultivars and landraces by utilizing the nearest neighbor clustering (Fig. 6e).

### Linkage disequilibrium (LD)

**Fig. 7:**
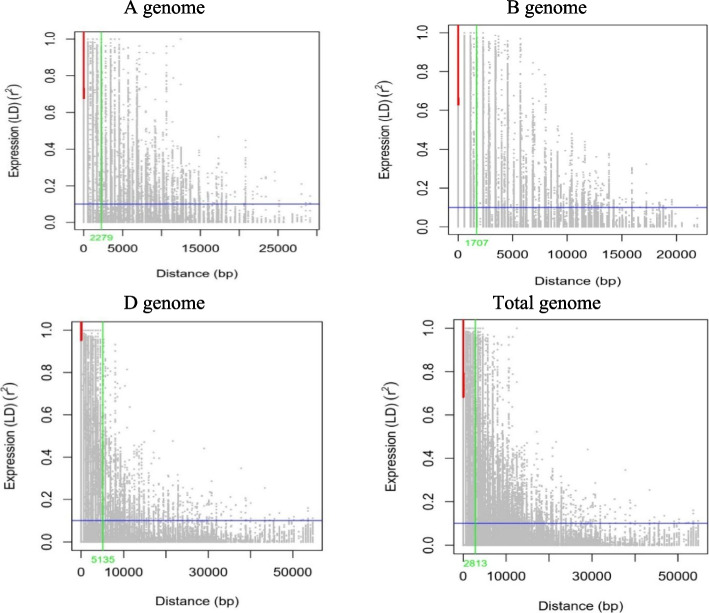
Overview of the linkage disequilibrium (LD) within the whole association panel per genome using imputed SNPs

The LD decreased with increased distances between SNPs and varied between and within chromosomes. There were 1,858,425 markers with *r*^2^ = 0.211 with varieties, out of which 700,991 (37.72%) had significant linkages at *P* < 0.001. The majority of significant marker pairs were located at a distance of < 10 cM, based on our observations. An analysis of landraces found 1,867,575 markers with *r*^2^ = 0.182, of which 847,725 (45.39%) displayed significant linkages at *P* < 0.001. Chromosome 4A marker pairs also demonstrated the strongest LD (*r*^2^ = 0.368). Most marker pairs with statistical significance were located at distances of < 10 cM. Genomes B and D had the most and least marker pairs (575,681 and 113,374), respectively (Fig. 7, Supplementary 1 Table 1).

### Single-nucleotide polymorphisms for agronomic traits and various drought tolerance indices

**Fig. 8:**
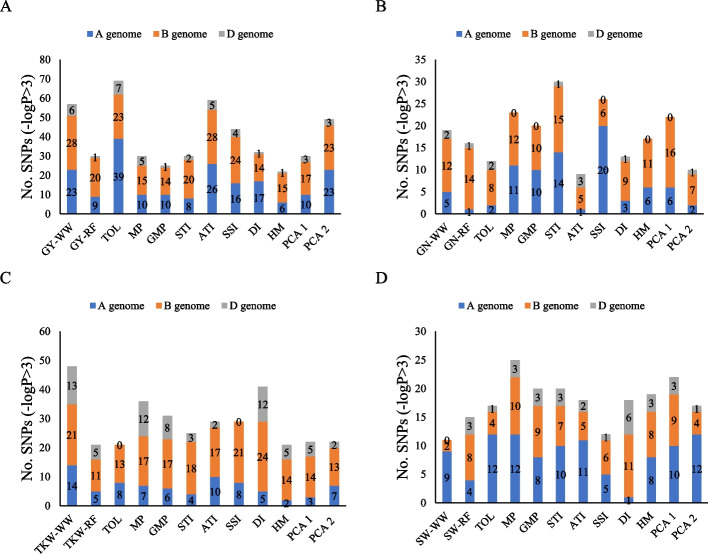
GWAS results (MLM method) for GY_WW_, GY_RF_, various drought tolerance indices, PCA1 and PCA2 (**A**), GN_WW_, GN_RF,_ various drought tolerance indices, PCA1 and PCA2 (**B**), TKW_WW_, TKW_RF_, various drought tolerance indices, PCA1 and PCA2 (**C**) and SW_WW_, SW_RF_, various drought tolerance indices, PCA1 and PCA2 (**D**) of Iranian landraces and cultivars wheat in well-watered and rain-fed environments. Abbreviations: GY, grain yield; GN, grains number per spike; TKW, thousand kernel weight; SW, spike weight; WW, well-watered; RF, rain-fed; TOL, tolerance index; MP, mean product; GMP, geometric mean product; STI, stress tolerance index; ATI, abiotic stress tolerance index; SSI, stress susceptibility index; DI, new drought resistance index; HM, harmonic mean; PCA, principal component analysis

**Fig. 9:**
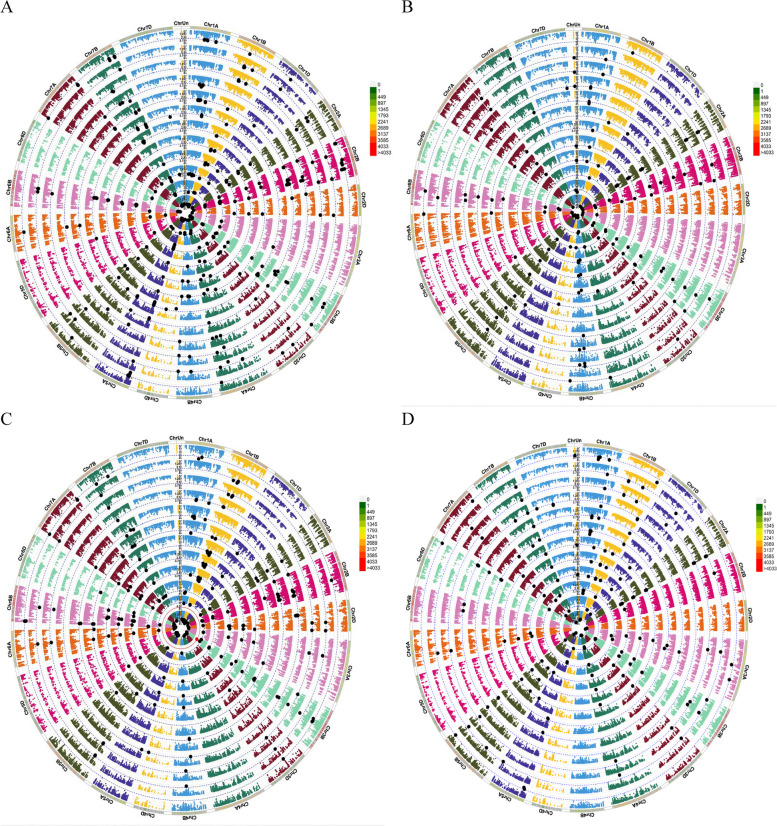
Circular Manhattan plots (MLM method) to draw common regions associated with A = GY_WW_, GY_RF_, various drought tolerance indices, PCA1 and PCA2, B = GN_WW_, GN_RF,_ various drought tolerance indices, PCA1 and PCA2, C = TKW_WW_, TKW_RF_, various drought tolerance indices, PCA1 and PCA2 and D = SW_WW_, SW_RF_, various drought tolerance indices, PCA1 and PCA2. Inner to outer circles represent average trait and breeding Values including Y_WW_, Y_RF_, TOL, MP, GMP, STI, ATI, SSI, DI, HM, PCA1 and PCA2, respectively. The chromosomes are plotted at the outmost circle where thin dotted blue and red lines indicate significant level at *P* value < 0.001 (− log10 (*p*) > 3) and < 0.00001 (− log10 (*p*) > 5), respectively. Green and red dots indicate genome-wide significantly associated SNPs at *P* value < 0.001 and < 0.00001 probability level, respectively. Scale between ChrUn and Chr1A indicates − log10 (*p*) values. Colored boxes outside on the top right side indicate SNP density across the genome where green to red indicates less dense to dense. Abbreviations: GY, grain yield; GN, grains number per spike; TKW, thousand kernel weight; SW, spike weight; WW, well-watered; RF, rain-fed; TOL, tolerance index; MP, mean product; GMP, geometric mean product; STI, stress tolerance index; ATI, abiotic stress tolerance index; SSI, stress susceptibility index; DI, new drought resistance index; HM, harmonic mean; PCA, principal component analysis

In total, 477 significant marker pairs (MTAs) were identified for GY and related stress tolerance indices [–log10 *P* > 3] by using MLM approaches. Of the total number of MTAs in the MLM method, 197, 241, and 39 MTAs were assigned to genomes A, B, and D, respectively. Using the MLM method, the number of significant MTAs for GY_WW_, GY_RF_, TOL, MP, GMP, STI, ATI, SSI, DI, HM, PCA1, and PCA2 traits were 57, 30, 69, 30, 25, 30, 59, 44, 32, 22, 30, and 49, respectively (Fig. 8a). The number of significant SNPs based on GN, TKW, SW, and stress tolerance indices associated with them were 217, 346, and 214, respectively. Drought tolerance indices for GN, TKW, and SW traits led to the discovery of 81, 79, and 102 significant SNPs for genome A, as well as 125, 200, and 83 significant SNPs for genome B. Additionally, 11, 67, and 29 significant SNPs were found for genome D, respectively. GN, TKW, SW, and stress tolerance indices associated with them led to the discovery of 81, 79, and 102 significant SNPs for genome A. In addition, for genomes B and D, 125, 200, and 83 as well as 11, 67, and 29 significant SNPs were found, respectively (Fig. 8b,c,d). SNPs with *P* values < 10^–3^ and < 10^–5^ (black and red) are highlighted in the Manhattan circle plot (Fig. 9).

**Fig. 10:**
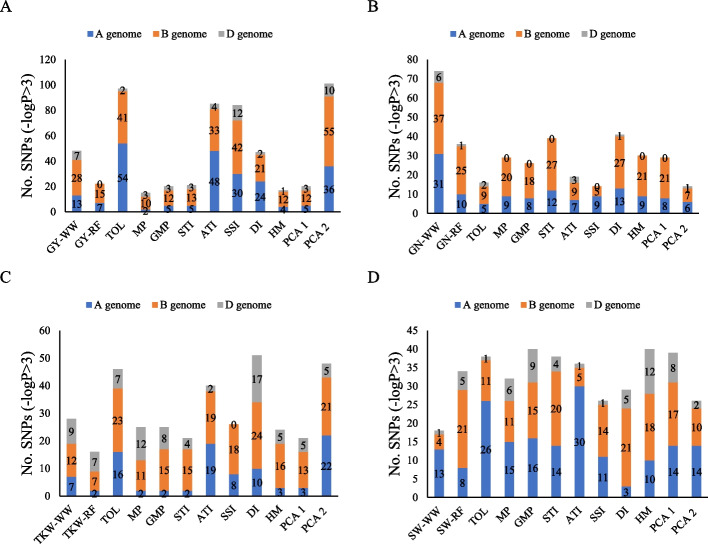
GWAS results (mrMLM method) for GY_WW_, GY_RF_, various drought tolerance indices, PCA1 and PCA2 (**A**), GN_WW_, GN_RF,_ various drought tolerance indices, PCA1 and PCA2 (**B**), TKW_WW_, TKW_RF_, various drought tolerance indices, PCA1 and PCA2 (**C**) and SW_WW_, SW_RF_, various drought tolerance indices, PCA1 and PCA2 (**D**) of Iranian landraces and cultivars wheat in well-watered and rain-fed environments. Abbreviations: GY, grain yield; GN, grains number per spike; TKW, thousand kernel weight; SW, spike weight; WW, well-watered; RF, rain-fed; TOL, tolerance index; MP, mean product; GMP, geometric mean product; STI, stress tolerance index; ATI, abiotic stress tolerance index; SSI, stress susceptibility index; DI, new drought resistance index; HM, harmonic mean; PCA, principal component analysis

**Fig. 11:**
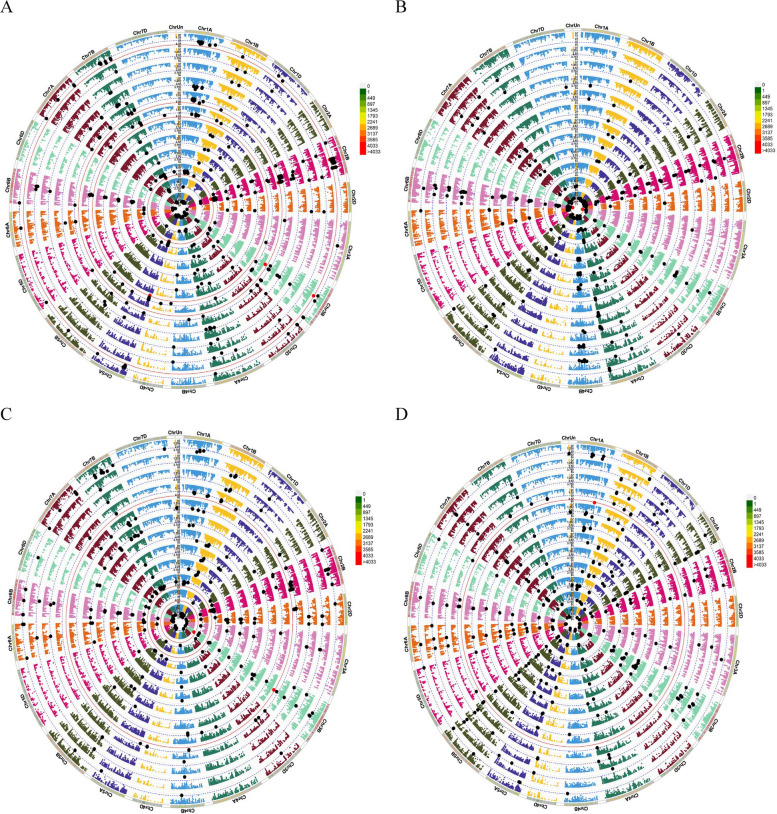
Circular Manhattan plots (mrMLM method) to draw common regions associated with A = GY_WW_, GY_RF_, various drought tolerance indices, PCA1 and PCA2, B = GN_WW_, GN_RF,_ various drought tolerance indices, PCA1 and PCA2, C = TKW_WW_, TKW_RF_, various drought tolerance indices, PCA1 and PCA2 and D = SW_WW_, SW_RF_, various drought tolerance indices, PCA1 and PCA2. Inner to outer circles represent average trait and breeding Values including Y_WW_, Y_RF_, TOL, MP, GMP, STI, ATI, SSI, DI, HM, PCA1 and PCA2, respectively. The chromosomes are plotted at the outmost circle where thin dotted blue and red lines indicate significant level at *P* value < 0.001 (− log10 (*p*) > 3) and < 0.00001 (− log10 (*p*) > 5), respectively. Green and red dots indicate genome-wide significantly associated SNPs at *P* value < 0.001 and < 0.00001 probability level, respectively. Scale between ChrUn and Chr1A indicates − log10 (*p*) values. Colored boxes outside on the top right side indicate SNP density across the genome where green to red indicates less dense to dense. Abbreviations: GY, grain yield; GN, grains number per spike; TKW, thousand kernel weight; SW, spike weight; WW, well-watered; RF, rain-fed; TOL, tolerance index; MP, mean product; GMP, geometric mean product; STI, stress tolerance index; ATI, abiotic stress tolerance index; SSI, stress susceptibility index; DI, new drought resistance index; HM, harmonic mean; PCA, principal component analysis

The mrMLM method discovered 233, 294, and 50 significant SNPs for GY_WW_, GY_RF_, DTIs, PCA1, and PCA2 in genomes A, B, and D, respectively. Further, GY_WW_, GY_RF_, TOL, MP, GMP, STI, ATI, SSI, DI, HM, PCA1, and PCA2 had 48, 22, 97, 15, 20, 21, 85, 84, 47, 17, 20, and 101 significant markers, respectively. The number of important SNPs based on GN, TKW, SW, and associated stress tolerance indices was 367, 371, and 396, respectively (Fig. 10). SNPs with *P* values < 10^–3^ and < 10^–5^ (black and red) are highlighted in the Manhattan circle plot (Fig. 11).

Using 43,525 SNPs and a significant value of –log10 (*P* > 5), MLM and mrMLM models identified a total of 67 and 24 MTAs for yield attributes and DTIs, respectively. There were a total of 26, 36, and 5 MTAs based on MLM, as well as 4, 20, and zero MTAs based on mrMLM, respectively, for genomes A, B, and D (Table 1).

**Table 1:** A summary of marker-trait associations for yield traits of Iranian wheat accessions

Genome	GY				GN				TKW				SW			
	mrMLM		MLM		mrMLM		MLM		mrMLM		MLM		mrMLM		MLM	
Significant	–log10 *P* > 3	–log10 *P* > 5	–log10 *P* > 3	–log10 *P* > 5	–log10 *P* > 3	–log10 *P* > 5	–log10 *P* > 3	–log10 *P* > 5	–log10 *P* > 3	–log10 *P* > 5	–log10 *P* > 3	–log10 *P* > 5	–log10 *P* > 3	–log10 *P* > 5	–log10 *P* > 3	–log10 *P* > 5
MTA	577	11	477	30	367	8	217	2	371	3	346	16	396	2	214	19
Genome A	233	2	197	13	127	1	81	0	96	0	79	0	174	1	102	13
Genome B	294	9	241	17	226	7	125	2	194	3	200	11	167	1	83	6
Genome D	50	0	39	0	14	0	11	0	81	0	67	5	55	0	29	0

*Abbreviations*: *GY* Grain yield, *GN* Grains number per spike, *TKW* Thousand kernel weight, *SW* Spike weight

### Gene ontology

**Fig. 12:**
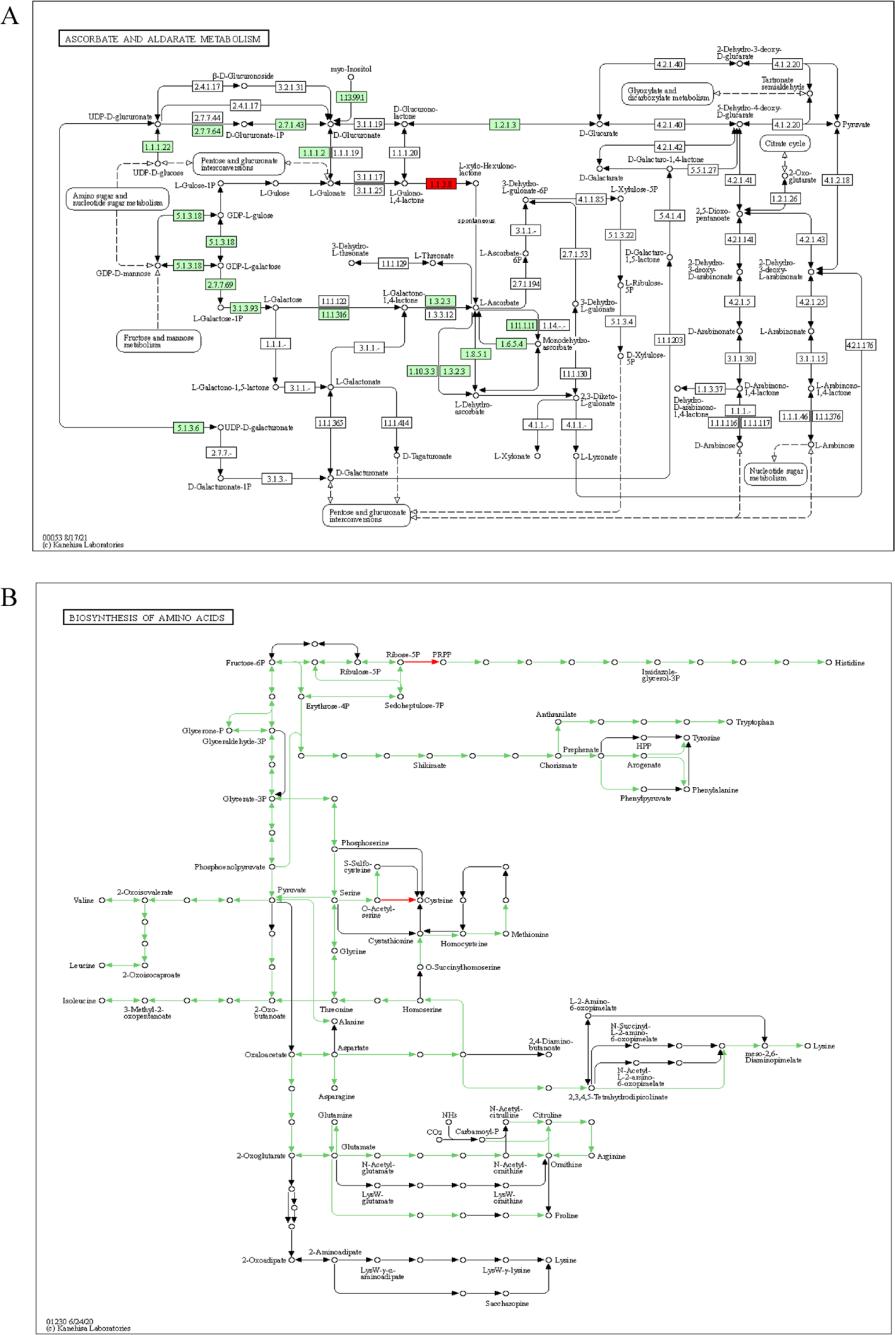
The KEGG pathway of ascorbate and aldarate metabolism (**A**) and pathway of biosynthesis of amino acids (**B**). The authors declare all that permissions were obtained for the appropriate copyright KEGG image depicted

The markers with the highest significance (*P* value < 0.0001) and pleiotropic impact were investigated thoroughly. Based on GO for DTIs for GN, TKW, and SW traits, 11, 6, 18, and 11 markers containing overlapping genes were identified, which are contained in important molecular and biological processes. Several biological and molecular processes were attributed to some of the discovered MTAs, including defense response, glycolytic process, lipid biosynthetic process, lipid metabolic process, fatty acid biosynthetic process, and response to wounding. The other processes were carbohydrate metabolic process, protein binding, ATP binding, nucleic acid binding, DNA binding, zinc ion binding, oxidoreductase activity, sulfotransferase activity, lipid binding, RNA binding and DNA binding (Table 2). Different pathways were found through rice reference genomes, including ascorbate and aldarate metabolism (Fig. 12a), biosynthesis of amino acids (Fig. 12b), oxidative phosphorylation (Supplementary 1 Fig. 1), fatty acid elongation (Supplementary 1 Fig. 2), and metabolic pathways (Supplementary 1 Fig. 3).

**Table 2:** Description of expected MTAs using imputed SNPs for tolerance indices of Iranian wheat accessions

No	Trait	SNP	Sequence	Trait- Index	Chromosome	Position (bp)	Gene ID	ological process	Cellular component	Molecular process
1	GN	rs18828	TGCAGCATAACCTCAAGGGTGTTGGACACTTAATTCACCTAAGGTACCTTGGTCTATCAGGTAC	ATI	1D	12,505	TraesCS1A02G026400	defense response	-	ADP binding
2		rs65348	TGCAGTTTTCCGATCGGATATGTCAGCGGCGTCGAGGACCATGCATGGATCGTTTAAAGGTGAT	SSI, DI	1A	44,512	TraesCS1A02G148300	-	-	protein binding
3		rs5402	TGCAGAGACTCCTTAGTCTTATTCAGCACATTTGGAGCCTTGGAAATAGCAATACCCACCTCAG	TOL, ATI, PCA2	1B	26,163	TraesCS1B02G047700	regulation of transcription, DNA-templated	-	double-stranded DNA binding
4		rs5109	TGCAGAGAAAAAGAGATGTATCATTCAGAGGCCAAACAAAAGTGAAGAGACAAATTTGATCACG	GN,MP, GMP,STI	2A	59,228	TraesCS2A02G251800	protein phosphorylation, signal transduction, response to nutrient, response to water deprivation, potassium ion import across plasma membrane, stomatal movement, phosphorylation, intracellular signal transduction	nucleus, cytosol, plasma membrane, plastid	nucleotide binding, protein kinase activity, protein serine/threonine kinase activity, ATP binding, kinase activity, transferase activity, protein serine kinase activity, protein threonine kinase activity
5		rs30934	TGCAGCGCGTGGGCATCGGCCACGTTCTAAATGTCACTGCCATGGTCGCTGCCGCACTGGTGGA	GN_WW_	4A	125,389	TraesCS4A02G440300	transmembrane transport	membrane	transmembrane transporter activity
6		rs56604	TGCAGTAGAAACCACCACTAGATGCAGTTATTCTAGTGAAACATCCGAGATCGGAAGAGCGGGA	STI	5D	7959	TraesCS5A02G014400	glycolytic process	phosphopyruvate hydratase complex	phosphopyruvate hydratase complex, phosphopyruvate hydratase activity
7	TKW	rs804	TGCAGAAATACACTCCTAATTTTACATGAACGCTACTCATATGGATTTATCCACACGACGCTAC	TKW_RF_, SSI,DI	1A	52,471	TraesCS1A02G312800	-	-	-
8		rs165	TGCAGAAAACTAAGATGCAGTTATTTTCTGAGTGATGCGTACTGTTAATTCGATCACAGGAGTT	DI	1B	27,300	TraesCS1B02G049300	DNA repair, protein ADP-ribosylation, response to oxidative stress, response to abscisic acid	nucleus, integral component of membrane	DNA binding, NAD + ADP-ribosyltransferase activity, zinc ion binding, NAD binding
9		rs15519	TGCAGCACTCTGCAAGAAAAACGTCAAAGTAAGAACCACCTACCCACATCTGCTCCAATTCAAA	TKW_WW_	1D	47,767	TraesCS1D02G147800	lipid biosynthetic process	integral component of membrane	iron ion binding, oxidoreductase activity
10		rs2355	TGCAGAAGTGCAGATAATCAGACAAGCCAGCGAAACCCTAAGCCCTCGACTAATCTGTTCGATC	SSI, DI	2B	0	TraesCS2D02G011200	positive regulation of transcription, DNA-templated	-	transcription coactivator activity, chromatin DNA binding
11		rs16199	TGCAGCAGCAACCACCACCATGGAAAGAGAGAGACAGAGACGGTGAGCTCCTCTGGACAGCGAG	DI	2D	28,183	TraesCS2D02G082700	lipid metabolic process	-	oxidoreductase activity, acting on paired donors, with oxidation of a pair of donors resulting in the reduction of molecular oxygen to two molecules of water
12		rs51900	TGCAGGGTGGGGGCGGAGAAAAAGGAGGAGGGGCGGCCGAGATCGGAAGAGCGGGATCACCGAC	TKW_RF_, DI	2D	28,183	TraesCS2D02G082900	vesicle-mediated transport	plasma membrane, integral component of membrane	protein binding
13		rs59777	TGCAGTCTTTCAGAAGTGCAGATGTAAACGTATTGCTATATCAGTGGTTTGAACTACATGGTAA	TKW_WW_, TOL, ATI	2D	58,883	TraesCS2D02G152500	protein ubiquitination, positive regulation of protein catabolic process	-	protein binding
14		rs16023	TGCAGCAGAGGTGGTTTGGAGGTTTGGTGGCGGCAGGATTCCCCTCCCGCGGGCGGCTCGGCTC	TKW_RF_, DI	3B	56,892	TraesCS3B02G373500	auxin-activated signaling pathway, transmembrane transport, intracellular auxin transport	membrane, integral component of membrane	-
15		rs60485	TGCAGTGCAGAAGGCGGCGTCCATCTGGTCCATGGCCTTGAGCTCGCCG	SSI	4B	34,449	TraesCS4B02G027000	-	-	-
16		rs23471	TGCAGCCCCATGGCTGGCCACTGCCCCGCCGACGCCACCTGCGGGTTTGGAGACGCCACCACGC	TKW_WW_	5A	58,225	TraesCS5A02G431100	-	-	RNA binding
17		rs31132	TGCAGCGCTCTCGGCGGTGACTCGTCGTCGCTCGGTGGCATCACCATCAACAAGACACGCGCGC	TKW_WW_	5A	58,225	TraesCS5A02G433100	lipid transport	-	lipid binding
18		rs44444	TGCAGGAGCTGAGCAACGAGGCCACAGCCGCCGCAGAAAAGGAGTCCCTGAACGGCACACTGGC	TKW_WW_, MP, STI	6B	92,187	TraesCS6A02G402500	ubiquitin-dependent protein catabolic process, protein deubiquitination	intracellular anatomical structure	thiol-dependent deubiquitinase
19		rs44444	TGCAGACACTAGTATCATTGGAAGCACAGGATGAGTCCGTTAGACAGTTGGGGGAGCTGAGGCA	K2STI	4A	61,015	TraesCS6B02G151700	-	-	ADP binding
20		rs37640	TGCAGCTCGTCATCACCGCTCGCCCGCCCGCGTGGATGCAGAAGTGCTCGAACGCCGTGCGGAA	PCA1,TKW_RF_, MP, GMP, STI, HM	6A	55,893	TraesCS6D02G274600	fatty acid biosynthetic process	membrane	acyltransferase activity, acyltransferase activity, transferring groups other than amino-acyl groups
21		rs9536	TGCAGATGGGGCGTTTCACGACGCTAGGCTATTGAGTGGAACTGGCAGTTTAGGGCTAAGCAGT	PCA2,TOL, SSI	7A	0	TraesCS7A02G020300	-		-
22		rs49562	TGCAGGGACAGAGCCACCATGCATGCCATTCTGCTGCACTGGGTCAGGCTCCACGTCCCCAACA	TKW_WW_	7B	45,510	TraesCS7B02G057400	-	membrane, integral component of membrane	ionotropic glutamate receptor activity, ligand-gated ion channel activity
23		rs30571	TGCAGCGCGAGGCGGCGGAAGGCCCTCTTGACCTCGCCCCTGGAGGCCCCCGAGATCGGAAGAG	PCA2, TOL, SSI	7B	94,660	TraesCS7B02G415000	-	-	-
24		rs36375	TGCAGCTCCAGGTACTGGACGATGTCGTGAGGGTTGCGGCGGCGGAGCGGGTGGTCAAGGCCAC	PCA2,TOL, ATI	7A	135,625	TraesCS7B02G498400	-	-	sulfotransferase activity
25	SW	rs49008	TGCAGGCTCTGGAAACTTCCCAGTGCTTCCCTCCATGGAACTCATGTTCTTCAATGAACTATTG	SW_RF_,MP, GMP, HM,PCA1	1B	21,612	TraesCS1B02G033900	response to wounding	-	serine-type endopeptidase inhibitor activity
26		rs57106	TGCAGTAGTATTAACTGAAGCCAAAGTCGTTTTTGTTGTTGTTGTGATGACAGGGGCAATGAGG	MP, SW_WW,_GMP, STI, HM,PCA1	2A	92,517	TraesCS2A02G570400	-	-	-
27		rs2179	TGCAGAAGGCATCTCAATTCAAGGCCCAGCCGATGTTACGGCGAGCTCCAATGATAAAGCCTAT	TOL, ATI, SSI,PCA2	3A	75,276	TraesCS3A02G405600	-	integral component of membrane	-
28		rs64448	TGCAGTTGTAATCTTCCATGGAATCCCAACAAGTTTAGAGCGTGTCGATTCGTGGTAGATGGAT	SW_RF_, MP, GMP, STI, HM,PCA1	3B	56,892	TraesCS3B02G373400	-	membrane, integral component of membrane	monooxygenase activity, iron ion binding, oxidoreductase activity, acting on paired donors, with incorporation or reduction of molecular oxygen, heme binding
29		rs61637	TGCAGTGGGCGCACGGATCCCTCAACGCGCTCTCCTGGGGCCTCCTGCTCCCCGTGGGCGCGGC	STI	4A	111,120	TraesCS4B02G265500	-	-	-
30		rs39277	TGCAGCTGCTGCTGGAGCAGCGGGTGCATCGCCGTCGCCGCCGAGATCGGAAGAGCGGGATCAC	SW_RF_	4A	111,179	TraesCS4D02G312700	-	-	-
31		rs55954	TGCAGTAATCTACTAGATACGGAGTACTATGGAAGTGAGGGAGCAGCCGCGCGCGCTTTTAATG	TOL, ATI, PCA2	5A	9093	TraesCS5A02G303400	-	-	oxidoreductase activity
32		rs36801	TGCAGCTCCGTGAGGCACACCGCGCACACGTCGTCGTCGCCGCCCGCGCCGCCGTCGTGGGCGC	ATI	6D	117,663	TraesCS6D02G389000	-	integral component of membrane	-
33		rs63819	TGCAGTTGACATGGTGGTTTTGTTCAAGGCACGGGCTCAAGAATCCAAAGAAACAAAATTTATT	DI	6D	117,663	TraesCS6D02G389600	-	integral component of membrane	aspartic-type endopeptidase activity
34		rs63548	TGCAGTTCGTCGTGGCGATGTTCGCCATGGACACGTGGCAATACTTCGTGCACAGGTATCTCCA	SW, TOL	7B	50,057	TraesCS7B02G092300	lipid biosynthetic process	integral component of membrane	iron ion binding, oxidoreductase activity
35		rs40986	TGCAGCTTCGGTTGGCCAGAGCCACACAACTCCACAAGTGAGCACATGCTAGCTGATGCATCCA	SW_RF_, DI	7B	51,193	TraesCS7B02G200200	-	-	-
36	GY	rs7126	TGCAGAGTCAGGCGGTAGGAAAAGGAGCGAAGGGGAACGGGGAACTCAGATCTGACGGGCAGCG	TOL	1A	46,787	TraesCS1B02G273100	regulation of transcription, DNA-templated	-	nucleic acid binding, DNA binding, zinc ion binding
37		rs6689	TGCAGAGGCCTGTTGACGTTGTGGTACAAGCCCGAGATCGGAAGAGCGGGATCACCGACTGCCC	SSI, PCA2	2B	7969	TraesCS2B02G029300	-	-	-
38		rs15903	TGCAGCAGAGAATAATAGATGGAGGGAGGGGTGGTGCAAGTATAGCACCCGAGATCGGAAGAGC	GY_RF_, GMP, STI, HM,PCA1	2B	34,160	TraesCS2B02G084300	-	-	ADP binding
39		rs7932	TGCAGATATTTATCGCCCAAGAGCAAAGATGCTTGACCAGGATTTGGATTGCGGACCGAGATCG	TOL, SSI, DI, PCA2	2B	86,479	TraesCS2B02G552400	transcription, DNA-templated	-	DNA binding, DNA-directed 5ʹ-3ʹ RNA polymerase activity, ribonucleoside binding
40		rs2997	TGCAGAATTGACAGATGCATCAAAATTGGTAGCCGCTGAAGCTAACAATGCTCATGTTGATGTT	TOL	3A	77,549	TraesCS3A02G417600	regulation of growth	-	
41		rs63419	TGCAGTTCGAGCGCCGATGGTGCCTCTTGTTGTGTTGTGTCCCCCCTCGCCATGTGTTGTCCAT	GY_RF_,TOL	4A	61,015	TraesCS4A02G156500	cation transport, calcium ion transport, transmembrane transport	integral component of membrane	cation transmembrane transporter activity, calcium:proton antiporter activity
42		rs51991	TGCAGGGTTCGCTCGTCGACGTCAACCCTTTGGAAGCGCAGCTCGAGCGCGGCATCCTTCTGGA	GY_RF_, DI	4A	129,369	TraesCS4A02G437000		-	ATP binding, ATPase
43		rs42463	TGCAGGAACTCCGCTCTGTCCTCGTCCTCGTCGTCGCTGTCGGCCTTGCTCGAGGTGCCGAGAT	TOL, ATI, PCA2	5A	9093	TraesCS5A02G295400	carbohydrate metabolic process	-	hydrolase activity, hydrolyzing O-glycosyl compounds
44		rs55429	TGCAGGTTTCAATTACGGAGGGAAAAACTCCAAGAAACTTATTGTTAGCAAGACGAAGTGACTG	GY_WW_	5A	109,694	TraesCS5A02G542100	-	-	protein binding
45		rs8046	TGCAGATCACTATGCTTGTATGGTGGATCTCTATGGCCGAGCTGGACTTATTGAAGAAGCACAC	ATI	6B	58,062	TraesCS6A02G338900	-	-	protein binding
46		rs24860	TGCAGCCGAGCTAATGGTGGTAGGGGTGGATGCTTTTTGTGAGCGACTGAGCGATGAGGAATGA	TOL	6B	1137	TraesCS6D02G008200	aromatic compound biosynthetic process, methylation	-	methyltransferase activity, O-methyltransferase activity, S-adenosylmethionine-dependent methyltransferase activity, transferase activity, protein dimerization activity

*Abbreviations*: *GY* Grain yield, *GN* Grains number per spike, *TKW* Thousand kernel weight, *SW* Spike weight, *TOL* Tolerance index, *MP* Mean product, *GMP* Geometric mean product, *STI* Stress tolerance index, *ATI* Abiotic stress tolerance index, *SSI* Stress susceptibility index, *DI* New drought resistance index, *HM* Harmonic mean

## Discussion

It is possible to examine how GY is affected under normal and drought conditions by using DTIs to select optimal genotypes. DTIs STI, GMP, MP, and HM are key indices for screening high-yielding genotypes in different moisture conditions [29, 30]. The previous study also demonstrated that STI is a useful parameter for distinguishing high-yield genotypes growing in both high-yielding and drought-tolerant environments [31–33]. The MP, GMP, STI, and HM parameters were related to GY. According to Ravari et al. [34] YSI, GMP, STI, and HM parameters were all well associated with the dependent variable for stress.

PCA and heatmap analyses have already been confirmed to be effective in identifying drought-tolerant and high-yield genotypes in tomato [35], chickpea [36], and switchgrass [37]. The MP, GMP, STI, and HM indices are effective for finding DT genotypes suitable for planting in a water-limited region according to the cluster analysis. Based on the PCA of Iranian cultivars and landraces, the first component could be linked to GY_WW_, GY_RF_, MP, STI, and GMP, and cultivars with high yields and DT could be identified as having this component. A similar finding was reported by Farshadfar et al. [38] for DTIs. According to our PCA results, GMP and HM were the most appropriate indices for screening in local varieties.

A high level of variation was uncovered in the studied traits for Iranian wheat accessions, suggesting the potential of the GWAS technique for exploring QTLs. A strong correlation between yield traits can be justified by indirect or direct contributions from other traits [39]. Regarding the wheat genome, the genetic areas responsible for such yield traits can be similar [40]. Mwadzingeni et al. [41], for example, discovered that a single locus influences numerous wheat traits such as plant height, spike length, and, grains per spike, all of which are connected frequently [42]. However, some loci affect only one crop attribute [41].

The frequency of the linked SNPs was higher in genome B than that of the other genomes. Because chromosome B is smaller than chromosome A, it appears that there is a clear association between chromosome size and SNP density [43, 44]. The increased frequency of SNP in the B genome was the result of evolutionary processes. Alipour et al. [45] and Mourad et al. [46] also reported this inference. Three separate subpopulations of wheat accessions were identified. Considering that wheat accessions have different pedigrees, this issue is expectable. There may be some relationships among accessions when common parents or origins exist in their pedigrees [40, 47].

Genomes D, A, and B have the highest LD, respectively. The strongest LD was recorded between marker pairs on chromosome 4A [48]. LD differences between genomes and accessions indicate the effects of breeding schedules in addition to evolutionary processes. In wheat Pakistan/China collections, Liu et al. [26] found that the distance of LD decay in native populations is less than that in cultivated varieties.

The number of GO-based on GY, GN, TKW, and SW were 11, 6, 18, and 11 markers containing overlapping genes were identified. Although only those connections with *P* < 0.0001 were considered significant, the remaining MTAs may be useful in improving wheat drought tolerance. These connections can be found in genomic areas that influence agronomic traits. Given that yield is a highly complex genetic trait with low heritability, MTAs for yield appeared significant at a higher *P* value. Most of the identified markers were located on chromosomes 4A, 5A, 7B, 1A, 1B, 6B, and 2B based on the studied traits and their related tolerance indices. A number of MTAs/QTLs have been found for GY in wheat chr. 7A [49–52], 7B [49, 52, 53], 3D [49], 3A [49, 52–54], 5B [49, 52, 55], 1B [49, 53, 56], and 2B [49, 53, 54, 56, 57]. MTAs/QTLs for TKW have been found in previous reports on chr. 7B [52], 7D [50], 5B [58], 3A [57, 58], 3B [50], 2D [56], 2A [50], 2B [50, 52, 57], and 1A [52, 56–58]. Therefore, MTAs on chr. 5A, 1B, 6B, and 1D are novel for TKW. As a result, MTA on chr. 4A and 5A have never been documented, and it is novel for wheat output.

The results of our study are in line with those of some studies made on the bread wheat of Iran, including Salarpour et al. [59], Salarpour et al. [8], Sobhanian et al. [60], Tahmasebi et al. [61], and Heidari et al. [62]. Tahmasebi et al. [61], stated that the most important QTLs for the thousand-grain weight, and GY were detected on chromosomes 1B, 1D-a, and 7D-b. In another study, Heydari et al. [62] reported that the major QTL located at the Hair–Xpsp2999 interval on chromosome 1A controlled the expression of grains/spike (*R*^2^ = 12.9% in 2004 and 22.4% in 2005), grain weight/spike (*R*^2^ = 21.4% in 2004 and 15.8% in 2005), and spike number (*R*^2^ = 15.6% in 2004 and 5.4% in 2005). The QTL for GY located on chromosomes 6A, 6B, and 6D totally accounted for 27.2% and 31.7% of the total variation in this trait in 2004 and 2005, respectively.

The flanking sequences of 43,525 SNPs were compared to RefSeq v2.0 and aligned accordingly. Surprisingly, the majority of marker pairs were found in the protein-coding areas, which regulate transcription. Other factors contributing to drought tolerance include DNA binding, transcription factor activity, and transmembrane transport. Some pathways were discovered using the rice reference genome, including ascorbate and aldarate metabolism, oxidative phosphorylation, biosynthesis of amino acids, fatty acid elongation, and metabolic pathways. In the metabolism of ascorbate and aldarate sucrose synthase, and sucrose-phosphate synthase are both genes that are involved in a metabolic pathway that is associated with DS tolerance [63]. Drought stimulates energy-intensive activities such as osmolyte production and oxidative phosphorylation, as well as increased respiratory rates [64, 65]. In oats [66] and wheat [67], fatty acid synthesis is beneficial in combating DS. The amino acid pathway is one of the amino acids produced by proline. The amino acid proline has been related to a number of osmoprotective properties that scavenge reactive oxygen species [68–71]. Under DS, drought-tolerant genotypes gained proline content faster and in higher proportions than sensitive equivalents, emphasizing its importance in drought-tolerance breeding. It has been discovered that genes that control proline content have cumulative effects [72, 73].

## Conclusion

MTAs are key elements for detecting genomic areas linked to agronomic traits in wheat under drought stress. The identified markers might be used to clone and fine map underlying genes, as well as perform gene introgression and marker-based selection in wheat under normal and drought conditions. The discovery of QTL-rich regions on Ch. 4A and 5A supports the theory that this chromosome is important for drought tolerance and should be utilized for wheat breeding. Furthermore, a large number of SNP correlations were discovered at the genome level on the B genome, which has been related to drought resistance. Further, the use of association mapping based on several drought tolerance indices can be highly effective in finding the most essential markers for drought tolerance as well as discovering linked gene networks.

## Materials and methods

### Experimental site

**Fig. 13:**
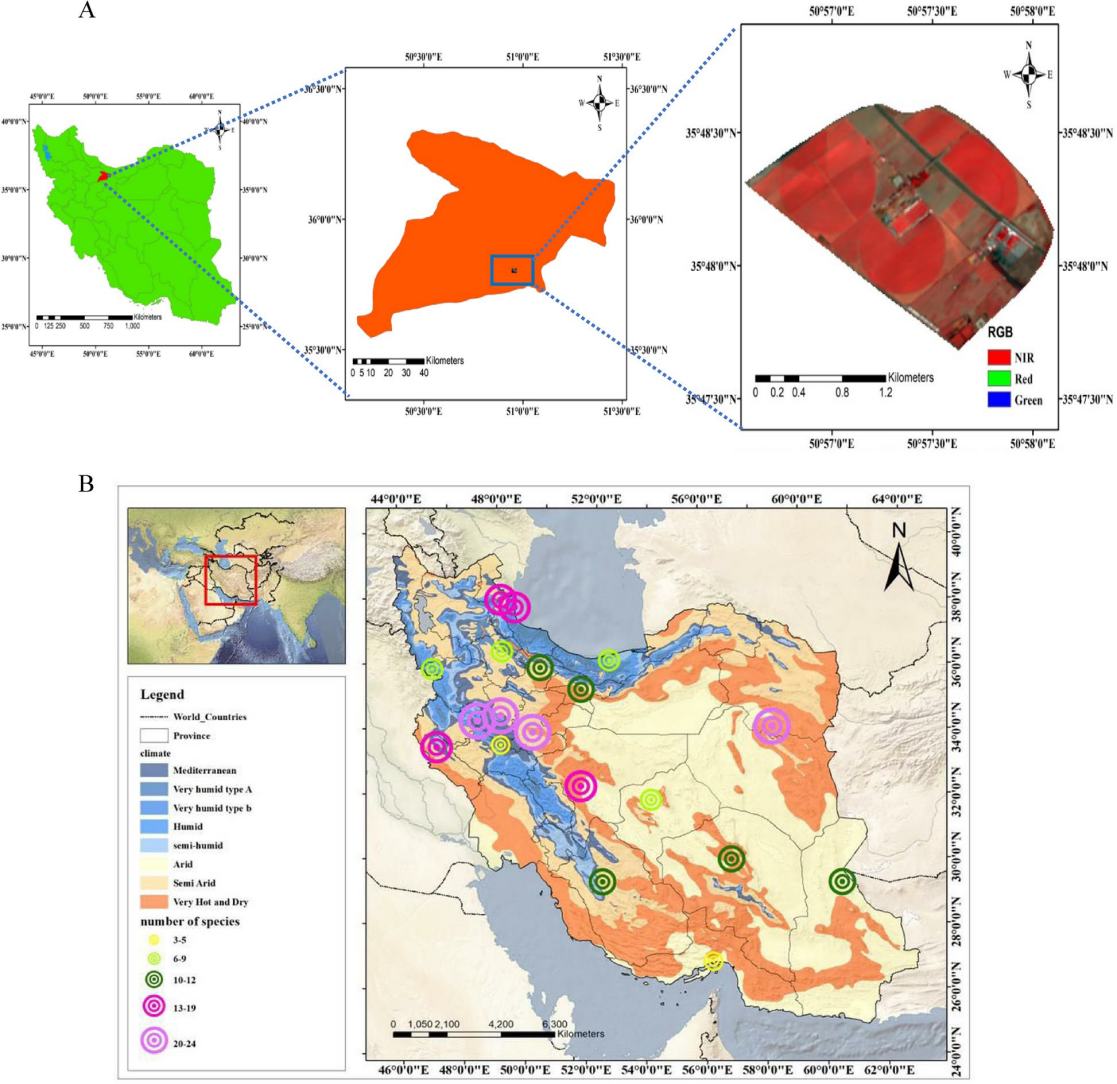
The geographical location of the study area (**a**) and the geographic distribution of Iranian wheat landraces collected between 1931 and 1968 years (**b**)

**Fig. 14:**
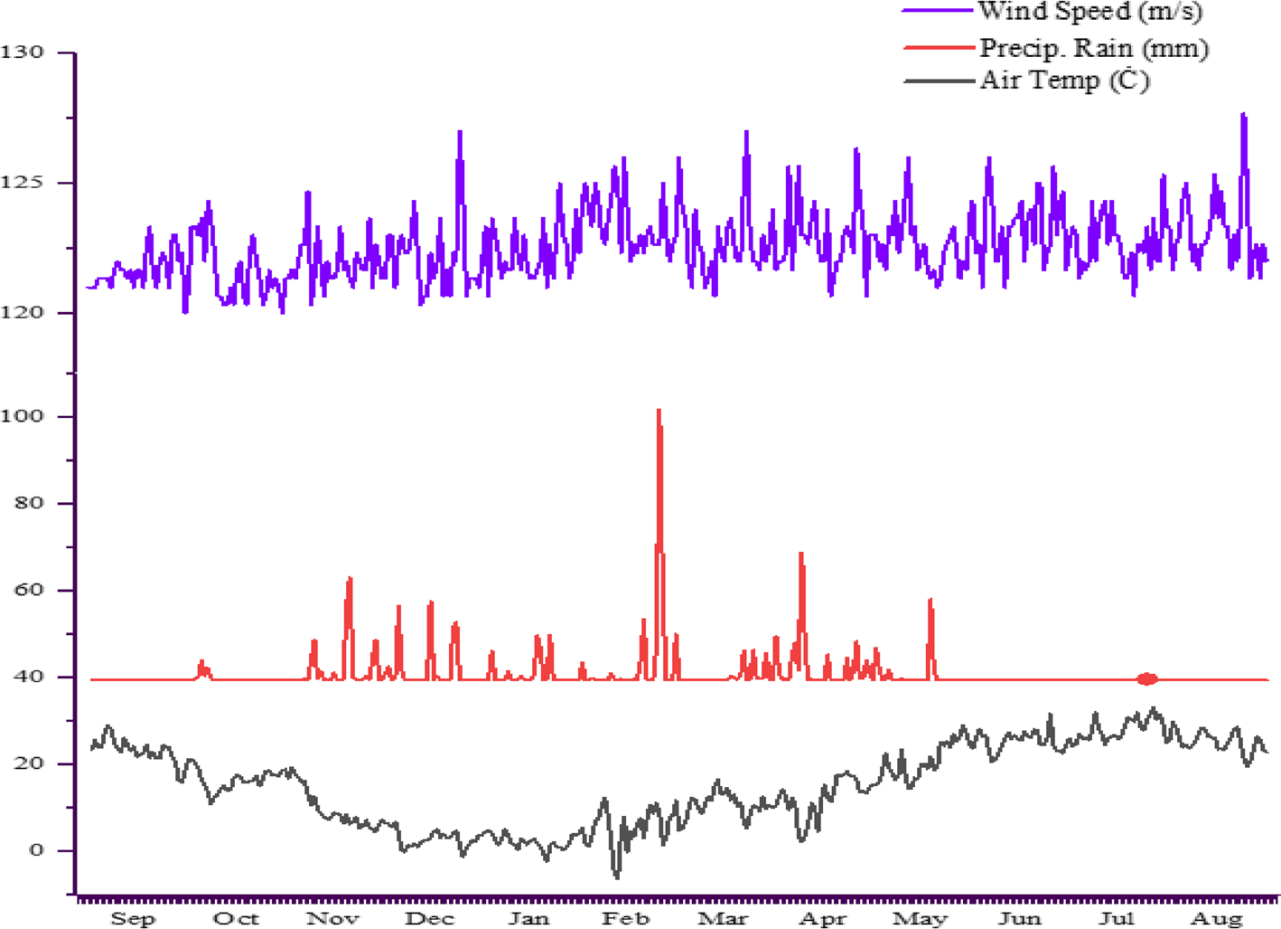
Climatic data in the studied environments

The research was conducted at the Agricultural and Natural Resources Campus of Tehran University. Figure 13 illustrates the geographical location of the study area (35°48′59′′N, 51°58′48′′E, 1321 m elevation) and the geographic distribution of Iranian wheat landraces collected between 1931 and 1968 years. Figure 14 shows the climatic characteristics of this field (Supplementary 1 Table 2). It covers approximately 246 ha and its main crops are barley, corn, wheat, and alfalfa. The climate in this region is dry and warm. A majority of the soil is clay and silt with an average annual temperature and precipitation of 22 °C and 248 mm.

### Plant materials and experimental design

This study followed 298 wheat genotypes collected from various regions and climates of Iran (Supplementary 1 Table 3) in alpha lattice design with two repeats during two crop seasons (2018–19 and 2019–20) under rain-fed (drought) and well-watered (normal) conditions. The plots consisted of four rows (1*1 m^2^) spaced 50 cm. The plant density was 300 plants/m^2^, and the sowing and harvesting dates in both years were November 1 and June 30. The threshold for irrigation implementation was determined based on 40 mm evaporation from an evaporation pan for well-watered crops. We used a crop coefficient of determination (*K*_*C*_) as well as a reference crop evapotranspiration equation, ET_0_ = E_pan_*K_pan_, where *E*_*pan*_ is the evaporation depth below the pan surface (40 mm), and *K*_*pan*_ denotes the pan coefficient (0.8) for each month, to determine evapotranspiration (ET_C_ = K_C_ * ET_0_) [2, 74]. In this study, the irrigation time was calculated by dividing the applied water for 1400 m^2^ (the cultivation area for 298 genotypes in two repeats) by the water discharge (10.8 m^3^/h). Water requirement (m^3^/ha) was estimated by multiplying the depth of ET_0_ (mm) by 10. Wheat is grown under the rain-fed regime and only receives rainfall as a source of water. Table 1 presents the patterns of rainfall during the cropping seasons. After physiological maturity, GY per plant was measured by isolating 20 plants and pounding their spikes, then weighing their seeds, which were weighed, followed by calculating the yield of a single plant. The traits measured in this study were GY (g per plant), spike weight (SW, gr), GN (per spike), and TKW (gr). The calculations of the DTIs were based on the trait yield for normal (Y_P_) and stress (Y_S_) conditions, and the total average trait (GY, GN, TKW, and SW) for normal (${\overline{Y} }_{\mathrm{P}}$) and stress (${\overline{Y} }_{\mathrm{s}}$) environments with the formulas listed in Table 3. Samples of plants are provided by the Gene Bank of Agronomy and Plant Breeding Group and these samples are available at USDA with USDA PI number (Supplementary 1 Table 3), respectively. The authors declare that all study complies with relevant institutional, national, and international guidelines and legislation for plant ethics in the methods section. The authors declare that all that permissions or licenses were obtained to collect the wheat plant.

**Table 3:** Drought tolerance indices used for investigation of Iranian wheat germplasm

Index	Abbreviation	Calculation formula	Reference
Tolerance index	TOL	$Yp-Ys$	[15]
Mean productivity	MP	$\frac{Yp+Ys }{2}$	[29]
Geometric mean productivity	GMP	$\sqrt{Yp \times { Y}_{S}}$	[30]
Stress tolerance index	STI	$\frac{Yp\times Ys }{{\overline{Y} }_{p}^{2}}$	[31]
Abiotic tolerance index	ATI	$\left[\frac{Yp-Ys}{\overline{Y }s / \overline{Y}p }\right]\times \left(\sqrt{Yp \times { Y}_{S}}\right)$	[38]
Harmonic mean	HM	$\frac{2\times Yp\times Ys }{Yp+{ Y}_{S}}$	[29]
Stress susceptibility index	SSI	$\frac{1-(Yp / Ys)}{1-(\overline{Y }s / \overline{Y }p)}$	[34]
Drought resistance index	DI	$\left[{Y}_{s}\left({Y}_{s}/{Y}_{p}\right)\right]/{\overline{Y} }_{s}$	[29]

### Genotyping-by-sequencing and imputation

In accordance with Alipour et al. [45], GBS libraries for Iranian wheat genotypes were established and sequenced. As reads were trimmed to 64bp and categorized into tags, SNPs were detected based on internal alignments, allowing for up to 3 bp of mismatch. The GBS pipeline was called Universal Network-Enabled Analysis Kit SNPs and discarded reads with a low-quality score (< 15). The imputation was performed with BEAGLE v3.3.2 and the w7984 reference genome [48]. Finally, SNPs with heterozygotes of < 10% and minor allele frequency greater than > 5% were considered for further analysis.

### Population structure and kinship matrix

STRUCTURE (version 2.3.4) was used to analyze the population structure of the landraces and cultivars of Iranian wheat [75]. This study employed a 30,000-step simulation phase, along with an admixture model, covering K = 1 to 10. The most likely number of sub-populations in this study was estimated by using ΔK. For association studies, Q-matrix was utilized as a structural matrix. Based on pairwise distance matrices counted in TASSEL [76], a neighbor-joining tree was formed and visualized using Archaeopteryx to explore the relationships between the cultivars and landraces of Iranian wheat.

### Genome-wide association study

Both MLM (mixed linear models) and mrMLM (multi-locus random-SNP-effect MLM) were applied to provide an unbiased estimation of marker effects. Using the MLM approach, it was possible to accurately associate marker traits between accessions and various MLM models for controlling both population structure (Q) and diffused associations (K) between accessions with the GAPIT package. In RStudio, GWAS was performed with the MLM and mrMLM using the GAPIT package [77]. The MLM approach considers accessions to be a random effect, the relevance of each is defined by a kinship matrix. The cluster analysis was conducted using kinship matrix elements as likeness measures, and the clusters were visualized by the unweighted double group approach with arithmetic mean (UPGMA) using a heat map. Moreover, –log10 (*P*) > 3 and –log10 (*P*) > 5 thresholds were used for statistically significant MTAs [78]. Confidence intervals for each chromosome were determined using LD decay [79]. A Manhattan plot was obtained by applying the CMplot package to explore associations between genotypes and phenotypes [80].

### Annotation of genes

The sequences surrounding all significantly associated SNPs were obtained by aligning them with IWGSC RefSeq v2.0 of the wheat 90 K SNP database used for Gramene (http://www.gramene.org/) gene annotation assessments. The identification of putative candidate genes was evaluated according to two parameters: a) being located in the vicinity of the peak marker, and b) having known functions and involvement in the studied traits in wheat (http://ensembl.gramene.Org; https://wheaturgi.versailles.inra.fr/SeqRepository/Annotations). Moreover, the significant SNPs were utilized in the enrichment analysis of gene ontology via KOBAS version 2.0 for testing in the KEGG. Finally, gene pathways were identified through the rice reference genome) Finally, gene pathways were identified through the rice reference genome ([80–82]; www.kegg.jp/kegg/kegg1.html).

### Statistical analysis

Descriptive statistics and correlation coefficients were obtained by R 4.1 using the ggplot2, dplyr, ggpubr, and psych packages to reveal the distribution of wheat traits. An analysis of heatmaps was performed in RStudio to classify wheat genotypes. Eventually, the evaluation and dispersion of wheat traits and genotypes across the biplot diagram were analyzed using PCA and the factoextra packages in RStudio.

### Supplementary Information

**Additional file 1: Supplementary Table 1.** A summary of LD observed among marker pairs and the number of significant marker pairs per genome and chromosome. **Supplementary Table 2.** Climatic data in the studied environments. **Supplementary Table 3.** Overview on the landraces and cultivars of Iranian wheat studied. **Supplementary Fig 1.** The KEGG pathway of oxidative phosphorylation. **Supplementary Fig 2.** The KEGG pathway of fatty acid elongation. **Supplementary Fig 3.** The KEGG pathway of metabolic pathways.

**Additional file 2.**

## Data Availability

The datasets generated and analyzed during the current study are available in Supplementary 2.

## Abbreviations

DS: Drought stress
GWAS: Genome-Wide Association Study
MTAs: Marker-trait associations
LD: Linkage disequilibrium
TKW: Thousand kernel weight
GN: Grains number per spike
SW: Spike weight
GY: Grain yield
WW: Well-watered
RF: Rain-fed
TOL: Tolerance index
MP: Mean productivity
GMP: Geometric mean productivity
STI: Stress tolerance index
ATI: Abiotic stress tolerance index
SSI: Stress susceptibility index
DI: New drought resistance index
HM: Harmonic mean
GBS: Genotyping-by-sequencing
MAF: Minor allele frequencies
PCA: Principal component analysis
