# Supplementary material for: Analysis of genetic diversity and genome-wide association study for drought tolerance related traits in Iranian bread wheat
Source: BMC Plant Biol. 2023 Sep 15;23:431. doi: 10.1186/s12870-023-04416-3 (PMC10503013; doi:10.1186/s12870-023-04416-3)
Supplement: Supplementary file 1 — Additional file 1: Supplementary Table 1. A summary of LD observed among marker pairs and the number of significant marker pairs per genome and chromosome. Supplementary Table 2. Climatic data in the studied environments. Supplementary Table 3. Overview on the landraces and cultivars of Iranian wheat studied. Supplementary Fig 1. The KEGG pathway of oxidative phosphorylation. Supplementary Fig 2. The KEGG pathway of fatty acid elongation. Supplementary Fig 3. The KEGG pathway of metabolic pathways. [file 12870_2023_4416_MOESM1_ESM.docx]

**Supplementary Table 1.** A summary of LD observed among marker pairs and the number of significant marker pairs per genome and chromosome.

| Chromosome | Total | | | |  | Landrace | | | |  | Cultivar | | | | |
| --- | --- | --- | --- | --- | --- | --- | --- | --- | --- | --- | --- | --- | --- | --- | --- |
|  | TNSP | r^2^ | Dis. (cM) | NSSP |  | TNSP | r^2^ | Dis. (cM) | NSSP |  | TNSP | r^2^ | Dis. (cM) | NSSP |  |
| 1A | 111575 | 0.111829 | 1.333712 | 49917 (44.74%) |  | 94575 | 0.116906 | 1.568634 | 34895 (36.9%) |  | 85625 | 0.148069 | 1.736676 | 27111 (31.66%) |  |
| 2A | 137150 | 0.251605 | 0.856962 | 79772 (58.16%) |  | 125450 | 0.289098 | 0.936772 | 68972 (54.98%) |  | 119450 | 0.288518 | 0.972951 | 57769 (48.36%) |  |
| 3A | 96450 | 0.130453 | 2.27878 | 44914 (46.57%) |  | 74950 | 0.134097 | 2.933748 | 28787 (38.41%) |  | 85000 | 0.15728 | 2.574908 | 25912 (30.48%) |  |
| 4A | 130500 | 0.317779 | 1.378513 | 79428 (60.86%) |  | 110850 | 0.369392 | 1.594492 | 66016 (59.55%) |  | 116700 | 0.36745 | 1.50704 | 58086 (49.77%) |  |
| 5A | 71850 | 0.132927 | 2.005721 | 32488 (45.22%) |  | 60100 | 0.146486 | 2.402626 | 24483 (40.74%) |  | 60600 | 0.166755 | 2.38547 | 18725 (30.9%) |  |
| 6A | 99050 | 0.158856 | 1.296073 | 52549 (53.05%) |  | 85850 | 0.178539 | 1.498357 | 40739 (47.45%) |  | 86550 | 0.178744 | 1.486057 | 29651 (34.26%) |  |
| 7A | 149700 | 0.193545 | 1.164988 | 78616 (52.52%) |  | 128550 | 0.211862 | 1.358487 | 64114 (49.87%) |  | 129900 | 0.232161 | 1.343972 | 49454 (38.07%) |  |
| 1B | 150800 | 0.154279 | 0.932852 | 80419 (53.33%) |  | 135600 | 0.154625 | 1.035051 | 64442 (47.52%) |  | 132400 | 0.20421 | 1.063407 | 49705 (37.54%) |  |
| 2B | 187300 | 0.156885 | 0.764253 | 102236 (54.58%) |  | 157350 | 0.176011 | 0.910909 | 79057 (50.24%) |  | 166950 | 0.19665 | 0.858127 | 66140 (39.62%) |  |
| 3B | 201700 | 0.210733 | 0.771726 | 119399 (59.2%) |  | 173200 | 0.220043 | 0.89872 | 90266 (52.12%) |  | 177550 | 0.243607 | 0.876084 | 78180 (44.03%) |  |
| 4B | 60050 | 0.115027 | 2.20477 | 23537 (39.2%) |  | 44800 | 0.09777 | 2.968273 | 12423 (27.73%) |  | 52600 | 0.142347 | 2.516753 | 13477 (25.62%) |  |
| 5B | 152400 | 0.15014 | 1.292476 | 80669 (52.93%) |  | 136300 | 0.14202 | 1.445522 | 57252 (42%) |  | 135650 | 0.202818 | 1.431617 | 55651 (41.03%) |  |
| 6B | 190850 | 0.13708 | 0.658245 | 99314 (52.04%) |  | 167500 | 0.135522 | 0.750676 | 71975 (42.97%) |  | 159700 | 0.203568 | 0.787671 | 66038 (41.35%) |  |
| 7B | 150100 | 0.121987 | 0.987127 | 70107 (46.71%) |  | 127550 | 0.12878 | 1.153868 | 51602 (40.46%) |  | 134150 | 0.155388 | 1.102364 | 41168 (30.69%) |  |
| 1D | 48650 | 0.238268 | 3.477302 | 26009 (53.46%) |  | 42500 | 0.226198 | 3.808863 | 20075 (47.24%) |  | 38350 | 0.285881 | 4.409069 | 16564 (43.19%) |  |
| 2D | 69550 | 0.183692 | 1.586178 | 31547 (45.36%) |  | 55400 | 0.163933 | 1.999469 | 21117 (38.12%) |  | 49600 | 0.228564 | 2.23156 | 16357 (32.98%) |  |
| 3D | 37050 | 0.116765 | 4.639072 | 5460 (14.74%) |  | 31800 | 0.165445 | 5.245984 | 11619 (36.54%) |  | 26800 | 0.137566 | 6.273779 | 5458 (20.37%) |  |
| 4D | 13500 | 0.122822 | 9.104484 | 4560 (33.78%) |  | 11800 | 0.130958 | 10.56137 | 3577 (30.31%) |  | 11550 | 0.154924 | 10.56621 | 2312 (20.02%) |  |
| 5D | 31750 | 0.130873 | 6.894582 | 12308 (38.77%) |  | 26250 | 0.134737 | 8.311197 | 9238 (35.19%) |  | 23700 | 0.147915 | 9.317761 | 5518 (23.28%) |  |
| 6D | 38300 | 0.123729 | 4.134238 | 15652 (40.87%) |  | 34900 | 0.136001 | 4.545476 | 12619 (36.16%) |  | 29750 | 0.137805 | 5.369092 | 6852 (23.03%) |  |
| 7D | 46700 | 0.150286 | 4.409549 | 17838 (38.2%) |  | 42300 | 0.147515 | 4.882439 | 14457 (34.18%) |  | 35850 | 0.201644 | 5.778975 | 10863 (30.3%) |  |
| A genome | 796275 | 0.195029 | 1.397647 | 417684 (52.45%) |  | 680325 | 0.220024 | 1.631824 | 328006 (48.21%) |  | 683825 | 0.232699 | 1.61945 | 266708 (39%) |  |
| B genome | 1093200 | 0.154972 | 0.95375 | 575681 (52.66%) |  | 942300 | 0.1588 | 1.106081 | 427017 (45.32%) |  | 959000 | 0.199661 | 1.084318 | 370359 (38.62%) |  |
| D genome | 285500 | 0.162046 | 4.054108 | 113374 (39.71%) |  | 244950 | 0.1634 | 4.684331 | 92702 (37.85%) |  | 215600 | 0.197637 | 5.369609 | 63924 (29.65%) |  |
| Whole genomes | 2174975 | 0.170566 | 1.523235 | 1106739 (50.89%) |  | 1867575 | 0.181706 | 1.766921 | 847725 (45.39%) |  | 1858425 | 0.211583 | 1.778371 | 700991 (37.72%) |  |

Abbreviations: r^2^: average squared allele frequency correlation; TNSP: Total number of SNP pairs; NSSP: Number of significant SNP pairs (P<0.001); Dis: Distance.

**Supplementary Table 2.** Climatic data in the studied environments.

| Year | Month | Max Temperature ⁰C | Min Temperature ⁰C | Average Temperature ⁰C | Total rainfall, mm | Average relative humidity | Sunny hours | Evaporation, mm |
| --- | --- | --- | --- | --- | --- | --- | --- | --- |
| 2017-2018 | November | 13.519 | 4.967 | 8.929 | 29.22 | 64.018 | 4.810 | 2.069 |
|  | December | 9.172 | -0.047 | 4.315 | 27.59 | 62.066 | 6.520 | 0.270 |
|  | January | 9.255 | -0.416 | 4.374 | 4.06 | 55.780 | 5.625 | 0.000 |
|  | February | 10.356 | -0.482 | 4.721 | 15.34 | 55.074 | 5.874 | 0.000 |
|  | March | 15.623 | 3.985 | 9.844 | 38.66 | 50.191 | 7.228 | 0.000 |
|  | April | 22.903 | 9.511 | 16.419 | 40.11 | 39.557 | 9.343 | 5.892 |
|  | May | 29.258 | 14.192 | 21.833 | 11.94 | 35.941 | 9.233 | 9.207 |
|  | June | 34.974 | 18.595 | 26.991 | 0.12 | 28.390 | 10.898 | 12.698 |
| 2018-2019 | November | 14.561 | 4.104 | 10.900 | 0.93 | 45.810 | 6.893 | 3.068 |
|  | December | 9.242 | -0.119 | 4.671 | 41.11 | 60.134 | 5.065 | 0.000 |
|  | January | 8.406 | -0.613 | 3.668 | 15.04 | 57.750 | 6.652 | 0.000 |
|  | February | 7.871 | -2.254 | 2.536 | 27.99 | 61.429 | 6.868 | 0.000 |
|  | March | 14.216 | 4.623 | 9.271 | 38.44 | 56.847 | 5.942 | 0.179 |
|  | April | 21.093 | 9.563 | 15.110 | 46.65 | 49.954 | 6.587 | 4.497 |
|  | May | 29.229 | 14.261 | 21.935 | 22.01 | 38.722 | 10.435 | 7.377 |
|  | June | 34.159 | 17.597 | 26.083 | 0.00 | 32.304 | 12.763 | 11.676 |
| 2019-2020 | November | 17.080 | 6.383 | 11.520 | 0.63 | 43.479 | 6.960 | 3.189 |
|  | December | 12.303 | 1.652 | 6.671 | 4.71 | 50.419 | 7.226 | 0.000 |
|  | January | 9.077 | -0.055 | 4.052 | 19.84 | 54.476 | 6.526 | 0.000 |
|  | February | 10.739 | 2.039 | 6.464 | 31.73 | 64.755 | 5.829 | 0.000 |
|  | March | 20.558 | 8.377 | 14.652 | 14.11 | 38.952 | 7.303 | 0.000 |
|  | April | 19.983 | 7.793 | 13.633 | 45.81 | 51.413 | 7.563 | 6.714 |
|  | May | 25.513 | 12.061 | 18.432 | 57.07 | 54.907 | 8.287 | 6.161 |
|  | June | 33.807 | 17.347 | 25.583 | 7.23 | 37.492 | 11.100 | 11.143 |

**Supplementary Table 3.** Overview on the landraces and cultivars of Iranian wheat studied

| Genetic background: Landraces | | | | | | |
| --- | --- | --- | --- | --- | --- | --- |
| No. | Region of origin (Province) | USDA_PI_NO |  | No. | Region of origin (Province) | USDA_PI_NO |
| 1 | Gilan | 625281 |  | 105 | Tehran | 621669 |
| 2 | Mazandaran | 625362 |  | 106 | Gazvin | 621704 |
| 3 | Khorasan | 625433 |  | 107 | Gazvin | 621706 |
| 4 | Khorasan | 625661 |  | 108 | Gazvin | 621712 |
| 5 | Khorasan | 625810 |  | 109 | Gazvin | 621716 |
| 6 | Kerman | 626156 |  | 110 | Azarbayjan-Gharbi | 620903 |
| 7 | Kerman | 626158 |  | 111 | Hamadan | 621420 |
| 8 | Kerman | 626215 |  | 112 | Hamadan | 621421 |
| 9 | Sistan-Balouchestan | 626223 |  | 113 | Bakhtaran | 621492 |
| 10 | Sistan-Balouchestan | 626226 |  | 114 | Hamadan | 621565 |
| 11 | Sistan-Balouchestan | 626234 |  | 115 | Mazandaran | 622084 |
| 12 | Markazi | 625080 |  | 116 | Gilan | 622098 |
| 13 | Markazi | 625081 |  | 117 | Gilan | 622099 |
| 14 | Markazi | 625123 |  | 118 | Gilan | 622105 |
| 15 | Markazi | 625127 |  | 119 | Mazandaran | 622247 |
| 16 | Markazi | 625139 |  | 120 | Mazandaran | 622264 |
| 17 | Mazandaran | 625263 |  | 121 | Mazandaran | 622272 |
| 18 | Sistan-Balouchestan | 626260 |  | 122 | Khorasan | 622311 |
| 19 | Sistan-Balouchestan | 626261 |  | 123 | Gazvin | 621717 |
| 20 | Esfahan | 626358 |  | 124 | Gazvin | 621735 |
| 21 | Esfahan | 626360 |  | 125 | Gazvin | 621736 |
| 22 | Esfahan | 626565 |  | 126 | Markazi | 621869 |
| 23 | Esfahan | 626566 |  | 127 | Markazi | 621908 |
| 24 | Esfahan | 626573 |  | 128 | Zanjan | 622063 |
| 25 | Ilam | 626699 |  | 129 | Yazd | 623109 |
| 26 | Hamadan | 626706 |  | 130 | Fars | 623123 |
| 27 | Khorasan | 626736 |  | 131 | Fars | 623125 |
| 28 | Yazd | 626747 |  | 132 | Fars | 623127 |
| 29 | Yazd | 626764 |  | 133 | Esfahan | 623008 |
| 30 | Khorasan | 626776 |  | 134 | Esfahan | 623069 |
| 31 | Esfahan | 626814 |  | 135 | Bakhtaran | 623090 |
| 32 | Esfahan | 626825 |  | 136 | Khorasan | 623091 |
| 33 | Yazd | 626846 |  | 137 | Khorasan | 622379 |
| 34 | Markazi | 626855 |  | 138 | Esfahan | 622894 |
| 35 | Fars | 626872 |  | 139 | Azarbayjan-Gharbi | 623266 |
| 36 | Kerman | 626908 |  | 140 | Bakhtaran | 623274 |
| 37 | Gilan | 626923 |  | 141 | Hamadan | 623291 |
| 38 | Gilan | 626924 |  | 142 | Yazd | 623318 |
| 39 | Hormozgan | 626932 |  | 143 | Fars | 623338 |
| 40 | Azarbayjan-Shargi | 626881 |  | 144 | Bakhtaran | 623344 |
| 41 | Fars | 626883 |  | 145 | Kordestan | 623345 |
| 42 | Azarbayjan-Shargi | 626895 |  | 146 | Kerman | 623377 |
| 43 | Azarbayjan-Shargi | 626904 |  | 147 | Kerman | 623379 |
| 44 | Zanjan | 627072 |  | 148 | Azarbayjan-Gharbi | 623136 |
| 45 | Khouzestan | 627099 |  | 149 | Fars | 623139 |
| 46 | Zanjan | 627102 |  | 150 | Azarbayjan-Gharbi | 623161 |
| 47 | Mazandaran | 627103 |  | 151 | Azarbayjan-Gharbi | 623162 |
| 48 | Khorasan | 627189 |  | 152 | Gilan | 623169 |
| 49 | Zanjan | 627055 |  | 153 | Khorasan | 623176 |
| 50 | Gilan | 627057 |  | 154 | Azarbayjan-Gharbi | 623510 |
| 51 | Markazi | 627061 |  | 155 | Bakhtaran | 623905 |
| 52 | Kerman | 627066 |  | 156 | Bakhtaran | 623908 |
| 53 | Hormozgan | 626933 |  | 157 | Bakhtaran | 623909 |
| 54 | Kerman | 626943 |  | 158 | Bakhtaran | 623953 |
| 55 | Azarbayjan-Gharbi | 626958 |  | 159 | Hamadan | 623980 |
| 56 | Esfahan | 626978 |  | 160 | Hamadan | 624215 |
| 57 | Khouzestan | 627036 |  | 161 | Ilam | 624240 |
| 58 | Khouzestan | 627038 |  | 162 | Ilam | 624251 |
| 59 | Azarbayjan-Gharbi | 627043 |  | 163 | Ilam | 623475 |
| 60 | Gilan | 627054 |  | 164 | Ilam | 623503 |
| 61 | Khorasan | 627236 |  | 165 | Bakhtaran | 623506 |
| 62 | Yazd | 627299 |  | 166 | Bakhtaran | 623507 |
| 63 | Hormozgan | 627356 |  | 167 | Bakhtaran | 623508 |
| 64 | Markazi | 627359 |  | 168 | Kerman | 623382 |
| 65 | Kerman | 627360 |  | 169 | Sistan-Balouchestan | 623417 |
| 66 | Bakhtaran | 627385 |  | 170 | Azarbayjan-Shargi | 623421 |
| 67 | Zanjan | 627399 |  | 171 | Azarbayjan-Shargi | 623428 |
| 68 | Azarbayjan-Shargi | 627410 |  | 172 | Ilam | 623473 |
| 69 | Bakhtaran | 627414 |  | 173 | Hamadan | 624596 |
| 70 | Bakhtaran | 627416 |  | 174 | Bakhtaran | 624804 |
| 71 | Bakhtaran | 627417 |  | 175 | Bakhtaran | 624805 |
| 72 | Hamadan | 627423 |  | 176 | Ilam | 624818 |
| 73 | Khorasan | 627460 |  | 177 | Ilam | 624837 |
| 74 | Yazd | 627484 |  | 178 | Ilam | 624838 |
| 75 | Azarbayjan-Shargi | 627787 |  | 179 | Ilam | 624846 |
| 76 | Kerman | 627842 |  | 180 | Ilam | 624849 |
| 77 | Sistan-Balouchestan | 627845 |  | 181 | Ilam | 624861 |
| 78 | Sistan-Balouchestan | 627849 |  | 182 | Kordestan | 624315 |
| 79 | Sistan-Balouchestan | 627852 |  | 183 | Bakhtaran | 624378 |
| 80 | Sistan-Balouchestan | 627853 |  | 184 | Bakhtaran | 624381 |
| 81 | Mazandaran | 627856 |  | 185 | Hamadan | 624576 |
| 82 | Zanjan | 627873 |  | 186 | Hamadan | 624580 |
| 83 | Esfahan | 627688 |  | 187 | Hamadan | 624582 |
| 84 | Yazd | 627723 |  | 188 | Hamadan | 624585 |
| 85 | Azarbayjan-Shargi | 627760 |  | 189 | Tehran | 624944 |
| 86 | Azarbayjan-Shargi | 627551 |  | 190 | Tehran | 624946 |
| 87 | Kordestan | 627587 |  | 191 | Tehran | 624947 |
| 88 | Esfahan | 627616 |  | 192 | Tehran | 624956 |
| 89 | Azarbayjan-Shargi | 627881 |  | 193 | Tehran | 624963 |
| 90 | Azarbayjan-Shargi | 627883 |  | 194 | Gazvin | 624980 |
| 91 | Mazandaran | 627905 |  | 195 | Gazvin | 624983 |
| 92 | Markazi | 627908 |  | 196 | Gazvin | 624985 |
| 93 | Markazi | 627948 |  | 197 | Gazvin | 624990 |
| 94 | Hamadan | 627963 |  | 198 | Markazi | 625047 |
| 95 | Zanjan | 627987 |  | 199 | Ilam | 624863 |
| 96 | Bakhtaran | 627990 |  | 200 | Ilam | 624864 |
| 97 | Bakhtaran | 628012 |  | 201 | Kordestan | 624894 |
| 98 | Mazandaran | 628084 |  | 202 | Kordestan | 624900 |
| 99 | Markazi | 628088 |  | 203 | Kordestan | 624901 |
| 100 | Esfahan | 628114 |  | 204 | Hamadan | 624910 |
| 101 | Ilam | 628189 |  | 205 | Hamadan | 624911 |
| 102 | Kordestan | 621619 |  | 206 | Hamadan | 624925 |
| 103 | Tehran | 621650 |  | 207 | Tehran | 624939 |
| 104 | Tehran | 621668 |  | 208 | Tehran | 624941 |


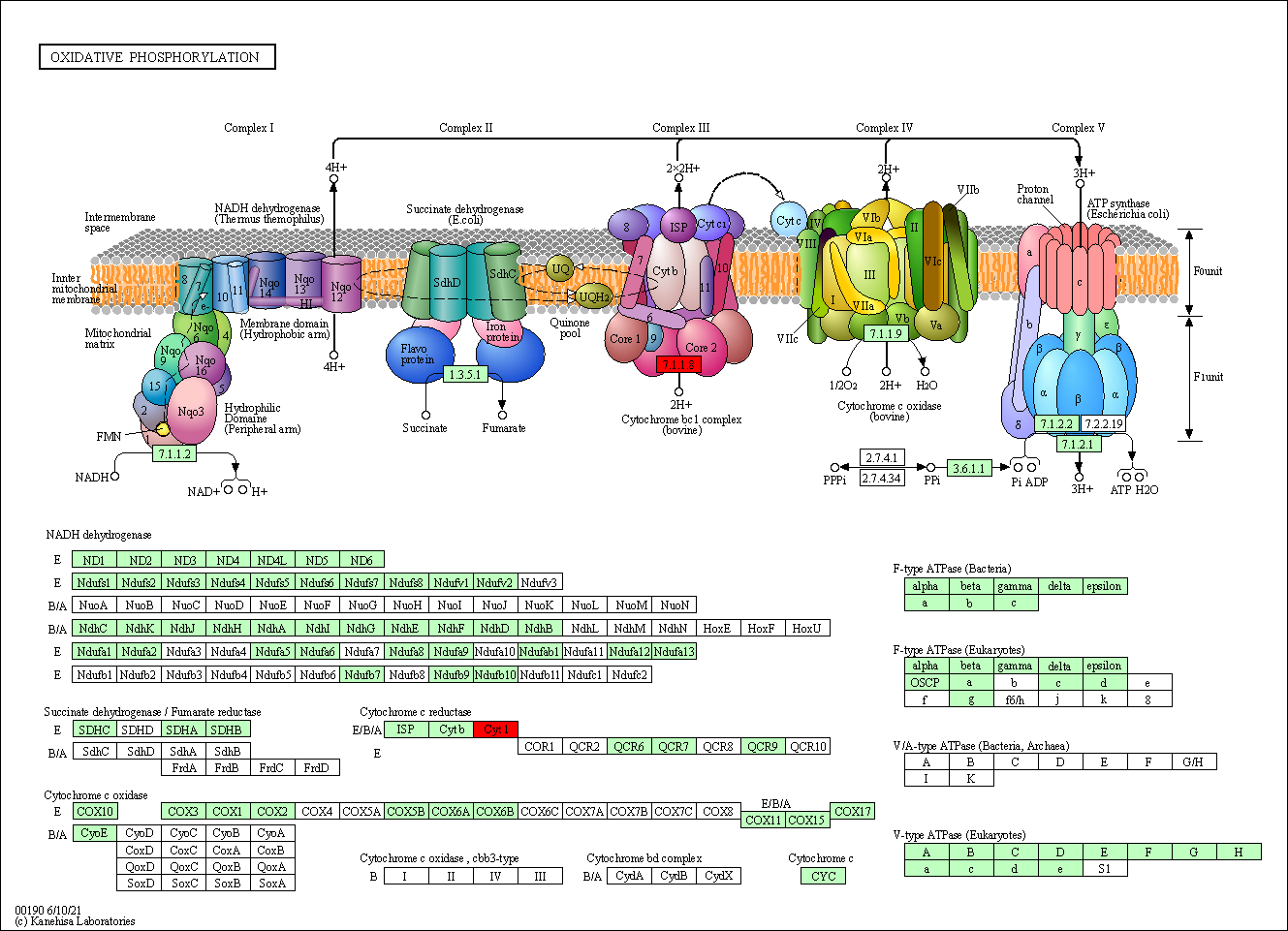


**Supplementary Fig 1.** The KEGG pathway of oxidative phosphorylation.


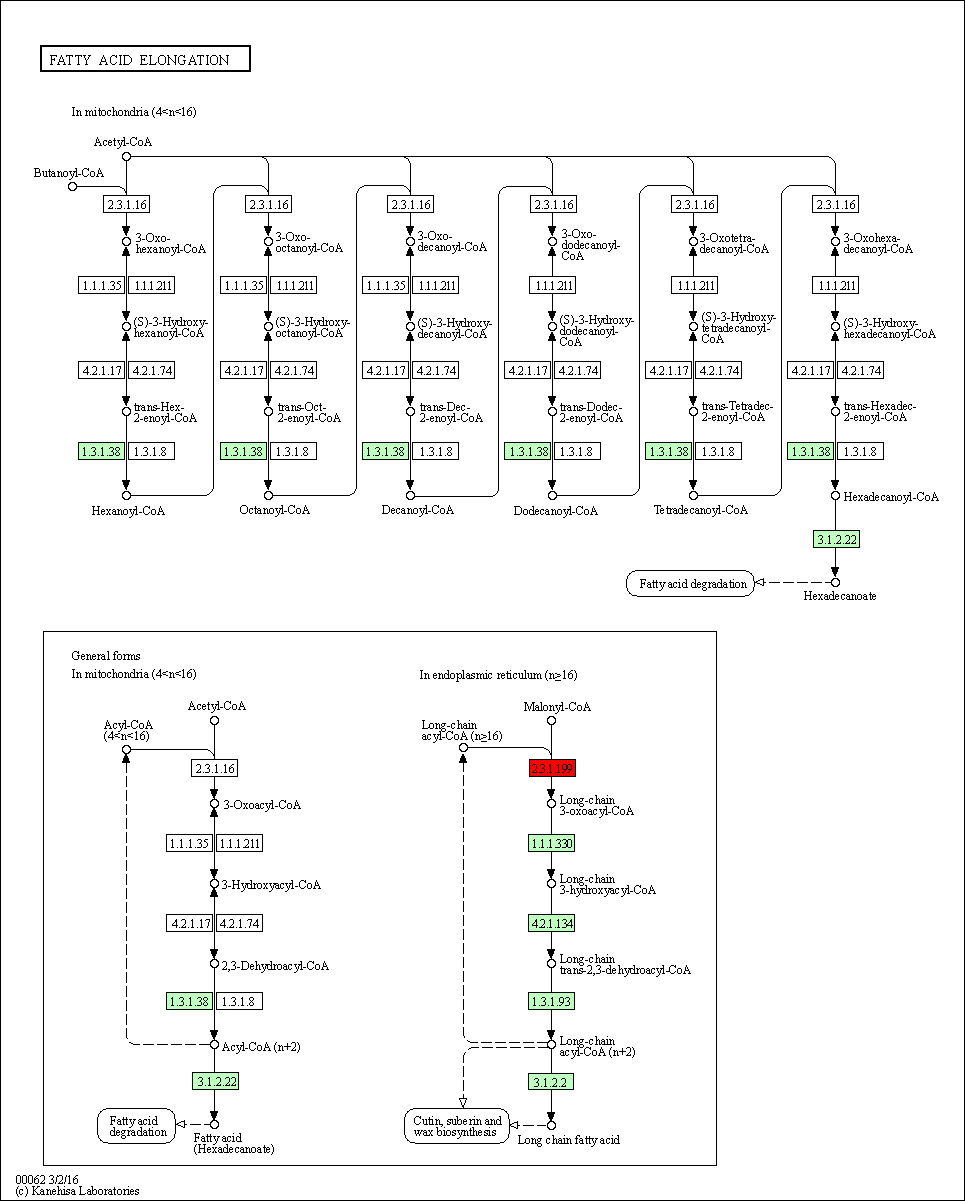


**Supplementary Fig 2.** The KEGG pathway of fatty acid elongation.


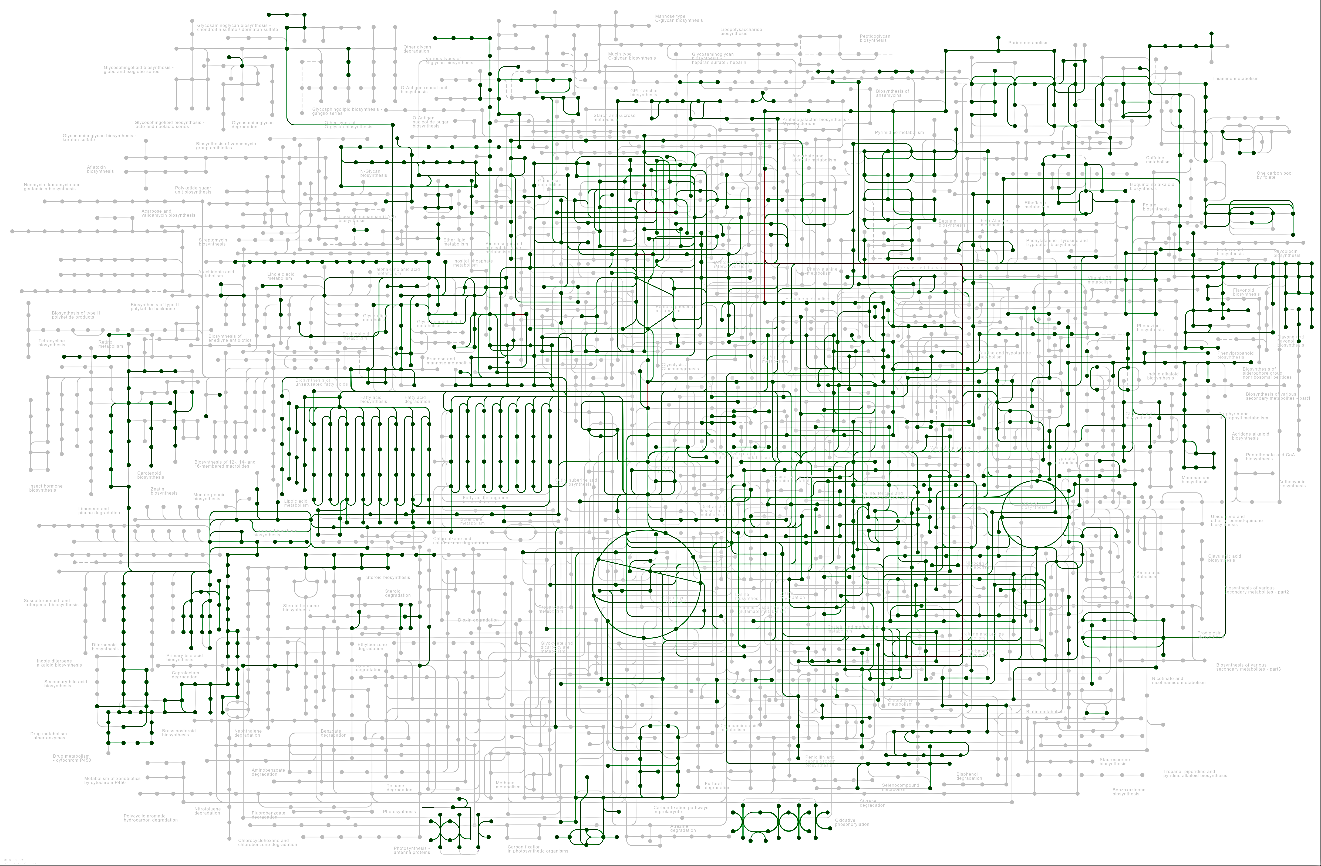


**Supplementary Fig 3.** The KEGG pathway of metabolic pathways.

The pathway map without coloring is the original version that is manually drawn by in-house software called KegSketch. The other pathway maps with coloring are all computationally generated as summarized below.

Reference pathway: this is the original version; white boxes are hyperlinked to KO, ENZYME, and REACTION entries in metabolic pathways; they are hyperlinked to KO entries in non-metabolic pathways.

Reference pathway (KO): blue boxes are hyperlinked to KO entries that are selected from the original version.

Reference pathway (EC): blue boxes are hyperlinked to ENZYME entries that are selected from the original version.

Reference pathway (Reaction): blue boxes are hyperlinked to REACTION entries that are selected from the original version.

Organism-specific pathway: green boxes are hyperlinked to GENES entries by converting K numbers (KO identifiers) to gene identifiers in the reference pathway, indicating the presence of genes in the genome and also the completeness of the pathway.
